# Diabetes-Induced Reactive Oxygen Species: Mechanism of Their Generation and Role in Renal Injury

**DOI:** 10.1155/2017/8379327

**Published:** 2017-01-09

**Authors:** Selim Fakhruddin, Wael Alanazi, Keith E. Jackson

**Affiliations:** Department of Basic Pharmaceutical Sciences, School of Pharmacy, University of Louisiana at Monroe (ULM), Pharmacy Building, 1800 Bienville Dr., Monroe, LA 71201, USA

## Abstract

Diabetes induces the onset and progression of renal injury through causing hemodynamic dysregulation along with abnormal morphological and functional nephron changes. The most important event that precedes renal injury is an increase in permeability of plasma proteins such as albumin through a damaged glomerular filtration barrier resulting in excessive urinary albumin excretion (UAE). Moreover, once enhanced UAE begins, it may advance renal injury from progression of abnormal renal hemodynamics, increased glomerular basement membrane (GBM) thickness, mesangial expansion, extracellular matrix accumulation, and glomerulosclerosis to eventual end-stage renal damage. Interestingly, all these pathological changes are predominantly driven by diabetes-induced reactive oxygen species (ROS) and abnormal downstream signaling molecules. In diabetic kidney, NADPH oxidase (enzymatic) and mitochondrial electron transport chain (nonenzymatic) are the prominent sources of ROS, which are believed to cause the onset of albuminuria followed by progression to renal damage through podocyte depletion. Chronic hyperglycemia and consequent ROS production can trigger abnormal signaling pathways involving diverse signaling mediators such as transcription factors, inflammatory cytokines, chemokines, and vasoactive substances. Persistently, increased expression and activation of these signaling molecules contribute to the irreversible functional and structural changes in the kidney resulting in critically decreased glomerular filtration rate leading to eventual renal failure.

## 1. Introduction

Diabetes is a group of chronic metabolic diseases marked by high plasma glucose levels (usually fasting plasma glucose (FPG) is ≥126 mg/dL) resulting from defects in insulin secretion or insulin action or both. The chronic hyperglycemia of diabetes induces several pathophysiological complications including cardiovascular abnormalities to renal failure. According to the American Diabetes Association [1], there are two main classes of diabetes: type 1 or insulin-dependent diabetes mellitus (IDDM) and type 2 or non-insulin-dependent diabetes mellitus (NIDDM). Type 1 diabetes is primarily caused by a cellular-mediated autoimmune destruction of *β*-cells of the pancreas and accounts for only 5–10% of diabetic cases. Conversely, type 2 diabetes is characterized by insulin resistance with relative insulin deficiency (i.e., patients secrete insulin but not enough to overcome the insulin resistance) and accounts for 90–95% of diagnosed diabetes cases.

The prevalence and incidence of diabetes and diabetic kidney diseases have alarmingly increased during recent decades. According to a 2014 national diabetes statistics report, 29.1 million United States citizens have diabetes which is 9.3% of the U.S. population. Every year, 1.4 million Americans are diagnosed with diabetes [2]. According to the 2014 World Health Organization (WHO) report, worldwide prevalence of diabetes was estimated to be 9% among adults of 18 years of age or older. In 2012, an estimated 1.5 million deaths were directly caused by diabetes [3]. WHO projects that diabetes will be the 7th leading cause of death by 2030 [4].

In line with the increasing incidence of diabetes, cases of chronic kidney disease (CKD) or end-stage renal damage (ESRD) have been growing significantly, since CKD is directly related to diabetes and/or hypertension. Approximately 1 of 3 adults with diabetes and 1 of 5 adults with high blood pressure have CKD. According to the 2014 National Chronic Kidney Disease Fact Sheet, more than 20 million adults (10% of all adults) have CKD in the United States. CKD is more prevalent in older people and most common among adults older than 70 years of age. It has also been observed that diabetes and hypertension are the leading causes of ESRD. In 2011, diabetes and hypertension were identified as the primary cause for 7 of 10 new United States cases of ESRD [5].

Diabetes-mediated chronic hyperglycemia evokes the onset and progression of renal injury because of its role in causing hemodynamic dysregulation along with abnormal morphological and functional nephron changes. The most important event that precedes renal injury is an increase in permeability of plasma proteins such as albumin through a damaged glomerular filtration barrier. This results in excessive urinary albumin excretion (UAE) through the nephron. Excess albumin excretion into urine is used as a prominent marker for diabetic kidney disease. Increased albumin leakage results from the impaired integrity of the glomerular filtration barrier (GFB), which is primarily responsible for retention of all plasma proteins. It is noted that GFB consists of three layers, where the visceral epithelial cells (podocytes) layer is highly vulnerable to ROS because of its nonproliferative nature even in response to injury [6, 7]. This results in early podocyte loss at the onset of diabetes and initiates increased protein excretion in urine.

Reactive oxygen species promote renal injury which eventually develops into chronic kidney disease. Diabetes-mediated ROS could be generated in both enzymatic and nonenzymatic pathways. Among many, NADPH oxidase (Nox) (enzymatic) and mitochondrial electron transport chain (mETC) (nonenzymatic) pathways are the prominent sources of ROS generation in the diabetic kidney and play a critical role in promoting pathophysiological events in kidney disease. In addition to NADPH oxidase (Nox) and mETC, other sources of ROS such as advanced glycation end products (AGEs) and uncoupled nitric oxide synthase (NOS) have been discussed in the current manuscript.

Hyperglycemia-induced ROS, particularly of Nox and mETC origin, are believed to cause the onset of albuminuria followed by progression of renal damage through podocyte depletion. ROS play a significant role in the onset of microalbuminuria through damaging the integrity of all the layers of the GFB. Once microalbuminuria occurs, ROS along with increased protein levels in the tubular ultrafiltrate can activate diverse aberrant signaling pathways to facilitate renal damage from the progressive stage to eventual end-stage renal damage (ESRD). Increased activation and/or production of various signaling mediators such as transcription factors, inflammatory agents, growth factors, cytokines, chemokines, and vasoactive molecules produce deleterious structural and functional glomerular alternations. These abnormal signaling cascades advance renal injury from progression of abnormal renal hemodynamics, increased glomerular basement membrane (GBM) thickness, mesangial expansion, extracellular matrix accumulation, interstitial fibrosis, and glomerulosclerosis to eventual end-stage renal damage. Though, at the outset, hyperglycemia-induced renal damage exhibits moderate structural and functional glomerular changes, such as hyperfiltration, untreated kidney develops most abnormal structural (Kimmelstiel-Wilson syndrome, nodular form of mesangial matrix) and functional (critically decreased filtration rate, <15–29 mL/min/1.73 m^2^) condition that warrants kidney dialysis. Albeit the role of glomerulus in progressive renal damage is substantial, tubular segment is not less important at all. Renal tubules rather increasingly contribute to the development of advanced stage of kidney damage which is beyond the scope of this review.

## 2. Basic Filtration Mechanism

Both kidneys receive about 22 percent of cardiac output that is equal to 1100 mL of blood in an adult. Blood flow into the glomerulus of the kidney is controlled by the afferent and efferent arterioles. The afferent arteriole drives the blood into the glomerulus, whereas efferent arteriole helps the blood flow out of the glomerulus into peritubular capillaries. Both of these arterioles can contribute to the filtration process by either facilitating blood flow to the glomerulus (via afferent arteriolar vasodilation) or increasing intraglomerular pressure (via efferent arteriolar vasoconstriction). Moreover, other physical factors play an important role in maintaining the net intraglomerular filtration pressure. Three critical pressures govern the filtration through the glomerular capillaries. They are (1) hydrostatic pressure inside the glomerular capillaries, also known as glomerular hydrostatic pressure (*P*_G_), which exerts 60 mmHg in favor of filtration, (2) hydrostatic pressure in Bowman's capsule (*P*_B_) that equals about 18 mmHg opposing the filtration, and (3) colloid osmotic pressure of the glomerular capillary plasma proteins, also known as glomerular oncotic pressure (*π*_G_), which shows 32 mmHg acting against the filtration. Hence, mathematically, the net filtration pressure can be calculated from *P*_G_−*P*_B_−*π*_G_  {*⁡*(60−18−32) mmHg*⁡*} which is equivalent to 10 mmHg. This net filtration pressure is maintained in the glomerulus at normal physiologic condition to promote filtration. Filtration of plasma fluid (45% of the total blood coming to the glomerular capillaries) results in easy permeation of relatively low molecular weight plasma components including electrolytes (Na^+^, K^+^, and Cl^−^), organic molecules (e.g., glucose, amino acids, and peptides), HCO_3_^−^, HPO_4_^−^, and all waste products such as urea, uric acid, and creatinine along with water into Bowman's space. The filtrate then flows along the renal tubule through which almost all essential components are reabsorbed except the waste products. In addition, it is very interesting to note that glomerular capillary wall is very efficient to retain larger molecules including plasma proteins during filtration, albeit a significant amount of protein can be permeated into the urinary space. The proteins that escape glomerular capillary barriers usually include *β*-2 microglobulin, immunoglobulin light chains, and small amounts of albumin. Most of these escaped proteins are reabsorbed and catabolized by the proximal tubular epithelium, thus further minimizing urinary excretion of protein content. For a normal adult, urinary protein excretion does not exceed 200 mg/day, of which very little (10–20 mg/day) is albumin [8–10]. However, structural and functional aberration of glomerular capillary barriers can lead to excess urinary excretion of proteins, abundantly albumin in different disease conditions including diabetes [11, 12], hypertension [13], and hyperlipidemia [14]. Excretion of proteins and excretion of albumin into urine are termed as “proteinuria” and “albuminuria,” respectively. Proteinuria and albuminuria are synonymously used in clinical practice, since albumin is usually the most abundant urinary protein in different renal diseases. Urinary albumin excretion over 24 hours is used as the “gold standard” to define different albuminuric conditions such as (1) normoalbuminuria that exhibits urinary albumin excretion (UAE) of <30 mg/day, (2) microalbuminuria (UAE range is 30–300 mg/day), and (3) macro/overt albuminuria (UAE > 300 mg/day). Urine collection over 24 hours is a cumbersome job which may lead to inaccurate measurement of truly excreted albumin. That is why clinicians now use albumin/creatinine ratio (mg/mmoL) in a spot urine sample to accurately and conveniently measure albumin that can literally represent albumin concentration in 24 h urine volume [15–17]. In medical literature, microalbuminuria is increasingly recognized as an important marker to characterize acute to chronic renal diseases [18]. Diabetic patients exhibit early microalbuminuria as a sign of onset of renal injury, progression of which can lead to macroalbuminuria with advanced pathological events ranging from decreased glomerular filtration rate (GFR) and glomerulosclerosis to eventual end-stage renal damage (ESRD). Before we review diabetes-induced pathological changes of glomerulus in detail, we will give an account on the contributory role of glomerular filtration barrier in fluid filtration and protein retention underscoring their structural and functional features.

## 3. Glomerular Filtration Barrier: Structural and Functional Role in Filtration

The glomerular filtration barrier (GFB) is recognized as a highly specialized ultrafiltration device that is capable of filtering large volumes of plasma fluids with a high permeability to water and small and midsized solutes in plasma, while efficiently retaining relatively larger macromolecules within the circulation. The barrier is composed of three layers: the innermost fenestrated vascular endothelium, the glomerular basement membrane, and the outermost podocyte layer (also called the glomerular visceral epithelial cells) [19]. All the layers, more or less, can provide the charge and size selectivity for macromolecules, usually plasma proteins, to prevent their easy passage into the urinary space [20, 21]. However, the exact locations for various selective functions of the barrier are still debatable.

### 3.1. The Endothelium

The endothelial layer is composed of unusually flattened endothelial cells with a height around the capillary loops of approximately 50–150 nm. Remarkably, endothelial cell bodies are completely perforated by open holes or fenestrae which constitute 20–50% of the entire endothelial surface. The fenestrae are usually round having a diameter of 40–100 nm which is similar in size to that found by Bearer et al. [22] in a study using quick-freeze and deep-etch method in rat kidneys. The abundantly fenestrated endothelium renders high permeability to water and small solutes in the glomeruli. Though larger fenestrae apparently seem to allow free passage of relatively smaller albumin (3.6 nm in diameter), it may not happen due to negatively charged endothelial surface layer (ESL). ESL mainly consists of plasma membrane-bound “glycocalyx” and a larger endothelial cell coat containing proteoglycans, glycoproteins, and plasma proteins [19, 22–24]. The glycocalyx is also composed of proteoglycans and glycoproteins (e.g., selectins, integrin, and members of the immunoglobulin superfamily) which can act as its backbone molecules. The glycoproteins contain acidic oligosaccharides and terminal sialic acids (SAs). Of note, various endothelial cell adhesion molecules which play an important role in cell recruitment during pathogenic condition are good examples of glycoproteins. The glycocalyx includes three families of cell adhesion molecules as glycoproteins: selectin, integrin, and immunoglobulin superfamily. Intercellular cell adhesion molecules (ICAM) and vascular cell adhesion molecules (VCAM) are the members of the immunoglobulin family. On the other hand, the proteoglycans exhibit a complex network of many proteins including glycosaminoglycan (GAG) chains, syndecans, glypicans, mimecan, perlecan, and biglycan. Syndecan is rich in heparan sulfate and chondroitin sulfate chains. Moreover, the glycosaminoglycan contains five chains, namely, heparan sulfate, chondroitin sulfate, dermatan sulfate, keratan sulfate, and hyaluronan (or hyaluronic acid). These highly sulfated chains together with sialic acids of glycoprotein give the glycocalyx a net negatively charged luminal surface [25, 26].

This negatively charged layer can selectively restrict access of negatively charged plasma proteins such as albumin to the endothelial cell membrane leading to limited filtration of albumin. This is manifested by a recent study, where the investigators infused hyaluronidase (ESL degrading enzyme) solution into right jugular vein of mice for 4 weeks and found significant decrease in ESL thickness resulting in increased albumin filtration [27]. Besides hyaluronidase, other high-molecular-weight enzymes, namely, heparinase and chondroitinase, are reported to decrease ESL thickness and charge density with subsequent increment in albumin clearance [28–30].

### 3.2. The Glomerular Basement Membrane (GBM)

Glomerular basement membrane is a gel-like layer interwoven between endothelium and epithelial layer. Electron microscopic study demonstrates that GBM is composed of inner, middle, and outer sublayers designated as lamina rara externa, lamina densa, and lamina rara interna, respectively [31]. All the layers form a tight fibrous meshwork comprising of collagen IV, laminin, and nidogen/entactin along with proteoglycan (i.e., agrin and perlecan) and glycoproteins. Collagen IV is a major skeletal component of GBM and plays an important role in maintaining its integrity. Mutation or loss of collagen IV may lead to progressive renal diseases including basement membrane thinning, podocyte foot process effacement, and Alport syndrome; the latter is manifested by increased ferritin permeability. However, mild proteinuria in patients with Alport syndrome indicates a minor role of this collagen in permselectivity of proteins. Laminin is also an important structural component that can maintain ultrastructure of podocytes by interacting with integrin of basal podocyte membrane. Additionally, laminin is thought to prevent easy permeation of plasma proteins, since its loss or mutation can cause excessive proteinuria [32–34]. Like glomerular endothelium, GBM contributes to charge selective restriction to the passage of polyanionic macromolecules due to its net negative charge imparted by sulfated chains of proteoglycans and sialyl as well as carboxyl groups of glycoproteins. Heparan sulfate proteoglycans are abundantly found in GBM, whereas others (chondroitin, keratan, and dermatan) are rare, but hyaluronic acid is present. On the other hand, agrin proteoglycan predominantly contributes to the total heparan sulfates of GBM, though perlecan may have a part [35, 36]. Based on these observations, it seems that GBM is critical for limited protein leakage as supported by the findings that removal of heparan sulfate by heparanase infusion or anti-heparan sulfate antibody treatment showed massive proteinuria and high GBM permeability. Moreover, heparan sulfate level of GBM was reported to be reduced in patients with both type 1 and type 2 diabetes showing remarkable proteinuria [37]. However, its restrictive protein filtration role has been debated as many studies found insignificant leakage of protein even after reduction of the heparan sulfate components through downregulation of their contributing proteoglycans agrin/perlecan [38–40].

### 3.3. The Podocytes

The podocytes are terminally differentiated visceral epithelial cells covering the outer surface of the glomerular capillaries and maintain the integrity of the kidney filter. They consist of a voluminous cell body, primary processes (arm-like projections coming from the cell body), and foot processes (numerous slender feet projected from primary processes). The cell body faces urinary space and gives rise to primary processes. Both processes are enriched in microtubules and intermediate filaments such as desmin and vimentin. The primary processes further elongate toward the capillary to make secondary/foot processes that contain an actin-based cytoskeleton. Besides actin, foot processes (FP) also contain other contractile proteins such as myosin, *α*-actinin, vinculin, and talin, which together help the podocytes get anchored to the basement membrane via mainly *α*3*β*1 integrin, whereas *α*- and *β*-dystroglycans linked with utrophin also assist in the attachment. The foot processes of podocytes form fine filtration slit by interdigitating with the foot processes of neighboring podocytes. The filtration slits are 30–40 nm in width and are bridged by a thin diaphragm, known as the slit diaphragm (SD). In addition to SD, foot processes can also contain two more membrane domains called apical/luminal and basal membrane domains. All the three membrane domains are structurally and functionally connected to FP actin cytoskeleton, thus giving a pivotal role to actin for podocyte function and dysfunction. Interference in any of these domains causes active reorganization of the actin filament from its parallel and contractile bundles into a dense network resulting in foot processes effacement [41, 42].

On the other hand, slit diaphragm plays a remarkable role in filtration by providing charge and size selective barrier to the macromolecules because of its architectural nature (physical sieve having pore size of ~3.8 nm, the same diameter of an albumin molecule [43, 44]) and various functional proteins. Slit diaphragm (also apical membrane) is lined with a thick coat composed of sialoglycoproteins, including podocalyxin and podoendin, imparting the net surface negative charges to the podocytes. These proteins can charge-selectively prevent filtration of plasma proteins. Additionally, the slit diaphragm consists of many proteins arranged one upon another along a vertical bar conforming to a zipper like structure. Studies of molecular genetics of the slit diaphragm have so far identified many proteins as its integral components, though the list is still growing. Several proteins such as ZO-1 (zonula occludens-1), nephrin, CD2AP (CD2-associated protein), FAT, P-cadherin, NEPH1 (nephrin-like protein-1), and podocin are expressed within the slit diaphragm. Synaptopodin, a novel podocyte marker, is an actin-associated protein, expressed in the foot process, and plays a role in the motility of the foot processes. All these proteins have important structural and functional roles as an integral part of the kidney filter. Though in-depth discussion on each of the podocyte proteins is beyond the scope of our review, the readers are referred to some exciting reviews for more understanding of the podocyte proteins and functions [42, 45, 46] However, we will later discuss injurious effect of reactive oxygen species on these proteins that trigger different pathological events during early to advanced renal damage in both type 1 and type 2 diabetes.

### 3.4. The Glomerular Mesangial Cells (GMCs)

Mesangial cells are smooth muscle-like pericytes located in the intercapillary regions of the glomerulus. Though the mesangial cells are not an integrated structural part of the glomerular capillary barrier in the kidney filter, their contribution to the fluid filtration cannot be underestimated. They, along with the capillary barrier, form a coordinated biochemical unit and control the filtration rate as they have the capacities of regulating filtration surface area, intraglomerular blood volume and filtration pressure, and hormone as well as growth factor secretion. Contracting (e.g., Ang II and vasopressin) and relaxing (i.e., ANP and NO) hormones secreted by GMC can control blood flow to the capillary loops via preferential constriction and dilation of efferent and afferent arterioles, respectively, thus maintaining constant glomerular filtration rate (GFR) [47]. GMC-secreting growth factors such as PDGF, FGF, EGF, and CTGF influence mesangial cell proliferation and matrix production. PDGF is very important for the generation and maintenance of the capillary loops. In addition, GMCs can also cleanse GBM to maintain its permeability feature and take up macromolecules entering into the matrix of the mesangium. In nutshell, GMCs and glomerular layers interplay to maintain the integrity and homeostasis of the glomerulus which has been supported by the evidence that mesangiolysis using toxin or antibodies against mesangial cell antigen results in proteinuria and glomerular abnormalities [48, 49].

## 4. Hyperglycemia-Induced ROS and Mechanisms of Their Generation

The term reactive oxygen species (ROS) can be defined as highly reactive oxygen-centered chemical species containing one or two unpaired electrons, where an unpaired electron is one that exists in an atomic or molecular orbital alone. The unpaired electron containing chemical species can also be called “free radicals.” In medical literature, the term “ROS” is used as a “collective term” to include both radicals and nonradicals, the latter being devoid of unpaired electron. So, ROS are classified into two categories: (1) oxygen-centered radicals and (2) oxygen-centered nonradicals. Oxygen-centered radicals include superoxide anion (^∙^O_2_^−^), hydroxyl radical (^*∙*^OH), alkoxyl radical (RO^*∙*^), and peroxyl radical (ROO^*∙*^). Oxygen-centered nonradicals are hydrogen peroxide (H_2_O_2_), singlet oxygen (^1^O_2_), and hypochlorous acids (HOCl). Unlike ROS, reactive nitrogen species (RNS) are nitrogen-centered radicals and nitrogen-centered nonradicals. The nitrogen-centered radicals include nitric oxide (NO^*∙*^) and nitrogen dioxide (NO_2_^∙^), whereas nitrogen-centered nonradicals are peroxynitrite (ONOO^−^), alkyl peroxynitrite (ROONO), nitroxyl anion (NO^−^), nitrous acid (HNO_2_), and so on [50].

High glucose-induced ROS can be generated by both enzymatic and nonenzymatic pathways. The enzymatic pathways include nicotinamide adenine dinucleotide phosphate oxidase (NADPH oxidase), uncoupling of nitric oxide synthase (NOS), cytochrome P-450 (CYTP450), cyclooxygenase (COX), lipoxygenase (LOX), xanthine oxidase, and myeloperoxidase (MPO). Conversely, the nonenzymatic pathways include mitochondrial electron transport chain (mETC) deficiencies, advanced glycation end products (AGEs), glucose autooxidation, transition-metal catalyzed Fenton reactions, and polyol (sorbitol) pathway [51–53]. Among these, we will discuss below the major ROS generating pathways, such as NADPH oxidase, uncoupled NOS, mETC, and AGEs that are increasingly involved in the pathogenesis of diabetic kidney diseases as demonstrated by many studies (Figure 2) [54–60].

### 4.1. NADPH Oxidase

NADPH oxidase is one of the principal sources of ROS production in hyperglycemic conditions of different organs including the kidney. NADPH oxidase is a respiratory burst enzyme that was initially identified in phagocytes in 1933. The enzyme is responsible for production of millimolar amounts of superoxide using cytosolic NADPH as substrate, and the superoxide or its downstream metabolite H_2_O_2_ can kill microorganisms in burst-dependent manner of phagocytes. Since its early detection in phagocytes, a growing body of scientific studies identified and cloned five major subunits constituting the enzyme, NADPH oxidase. They are membrane-bound flavocytochrome b_558_ forming subunits such as gp91^phox^ (also known as Nox2), p22^phox^, and cytosolic subunits that include p47^phox^, p67^phox^, and p40^phox^. Membrane-bound and cytosolic subunits are called catalytic and regulatory subunits, respectively. In addition, a small GTP-binding protein called Rac1 (in nonphagocytes) or Rac2 (in phagocytes) has been recognized as an essential cytosolic component for NADPH oxidase activation. Phagocytic gp91^phox^ (Nox2) subunit does have six different homologs including Nox1, Nox3, Nox4, Nox5, DUOX1, and DUOX2 to make a NOX family of 7 members [61]. In addition to phagocyte, NADPH oxidase has also been identified in other nonphagocytic cell types including fibroblasts, vascular smooth muscle cells, renal cells (podocyte and mesangial and proximal tubular cells), and endothelial cells [62–64]. However, the rate of superoxide production in these nonphagocytic cells by phagocyte-type NADPH oxidase is lower than that of neutrophils, implying the intrinsic functional difference of the enzyme in phagocytic and nonphagocytic cells. Additionally, above-mentioned phagocytic gp91^phox^ (Nox2) homologs can also be expressed in different tissues other than phagocytes with a variation in their abundance from tissue to tissue. For example, Nox4 is highly expressed in renal cells, whereas Nox1 is in the colon epithelium and vascular smooth muscle cells (VSMCs) [61, 65]. Though one homolog can be predominantly expressed in a particular cell type, other homologs of the Nox subunit may be expressed as well. NADPH oxidase can be activated in response to different stimuli, namely, pathogens, receptor agonists, and shear stress. Typical activation of the enzyme in phagocyte involves translocation of the cytosolic subunits to the plasma membrane to bind with the cytochrome b_558_. In doing so, p47^phox^ is first phosphorylated to get released from its autoinhibitory conformation and then recruits other cytosolic subunits (p67^phox^, p40^phox^, and Rac2) to make a cytosolic complex. This complex is then translocated to the membrane, where it binds with flavocytochrome b_558_ subunits to trigger transfer of electron from NADPH substrate to molecular O_2_, resulting in superoxide formation. p47^phox^ can be phosphorylated by different mediators including Ang II and c-Src. It is notable that p40^phox^ may not be essential for the enzyme activation, while other components paly crucial role in the enzyme activity with a variation in membrane-bound Nox homologs from tissue to tissue. For example, Nox2 is exclusively involved in phagocytic enzyme activation, where Nox1 is more upregulated in VSMC and Nox4 is expressed more in renal cells. Nox1-4 isoforms require p22^phox^ subunit for the enzyme activation while Nox5 and DUOX do not [66, 67]. Recently, Paclet et al. in a landmark study by isolating active forms of NADPH oxidase complex showed that translocation of cytosolic p47^phox^ and p67^phox^ subunits and GTPase Rac to plasma membrane and their subsequent binding with cytochrome b_558_ (gp91^phox^ and p22^phox^) are required to promote ROS generation by NADPH oxidase [68].

### 4.2. Mitochondrial ETC

Mitochondria are another potential source of ROS production in diabetic condition. However, there is a controversy as to which source of NADPH oxidase and mitochondria is predominantly contributing to ROS generation in diabetic condition, since some scientists identify the first [69, 70] to be more potential source, while others are in favor of the latter [71, 72]. Mitochondria play a pivotal role in maintaining intracellular energy homeostasis by producing ATP from ADP and inorganic phosphate molecule in oxidative phosphorylation pathway. Production of ATP results from two phases: oxidation of NADH (or FADH_2_) to donate electrons to mitochondrial electron transport chain (ETC) and phosphorylation of ADP to ATP, so named oxidative phosphorylation. It should be noted that the electron donating NADH and FADH_2_ come from two pathways: (1) glycolytic pathway which produces NADH and pyruvate from oxidation of intracellular glucose by the action of a series of enzymes and (2) mitochondrial Krebs cycle which oxidizes pyruvate derived from glycolysis to further produce NADH and FADH_2_. Both NADH and FADH_2_ act as high reducing equivalents for mitochondrial ETC. Mitochondrial ETC is located at the inner membrane and is mainly composed of four stationary enzyme complexes along with two mobile carriers of electrons such as ubiquinone (also known as coenzyme Q_10_, abbreviated as CoQ_10_) and cytochrome c. The complexes are complex I (NADH : ubiquinone oxidoreductase), complex II (succinate : ubiquinone oxidoreductase), complex III (ubiquinol : cytochrome c oxidoreductase), and complex IV (cytochrome c oxidase). In addition, an ATP synthesizing complex V (also known as ATP synthase) is located on the inner membrane. Electrons donated by NADH to complex I are transported by mobile ubiquinone to complex III. Ubiquinone can also receive electrons from succinate-derived FADH_2_ through complex II. Once the electrons reach complex III, its mobile cytochrome c carries the electrons to complex IV, which ultimately sends the electrons to O_2_ to reduce it and the reduced oxygen is combined with matrix H^+^ to form water. Each NADH or FADH_2_ donate two electrons to CoQ_10_ at a time and two electrons finally reduce half of molecular oxygen (1/2O_2_) to give H_2_O. During the transport of electrons along the chain, protons from mitochondrial matrix are pumped into inter membrane space using the free energy of the electron transfer. This increases H^+^ concentration in the intermembrane space, resulting in increased proton gradient across the inner membrane. The intermembrane protons can again enter into the matrix through ATP synthase which uses the potential energy derived from downward flow of protons for ATP synthesis and the entered protons may either combine with reduced oxygen at complex IV to form water or get pumped into outer space [73].

Any dysregulation in the coordinated transfer of the electrons by the enzyme complexes results in the leakage of electrons. The leaked electrons in turn reduce O_2_ to form superoxide (^∙^O_2_^−^) which undergoes dismutation by manganese superoxide dismutase (MnSOD) in the matrix and Cu, Zn-SOD in the inter membrane space to form H_2_O_2_. Though the major sites for electron leakage in mitochondrial ETC have been controversial, growing scientific evidence showed that complex I and complex III are the prominent sources of electron escape and ROS generation [72, 74–76].

Complex I generates superoxide (^∙^O_2_^−^) from ubiquinone-mediated electron leakage when large electrochemical proton gradient promotes reverse flow of electrons to complex I from downstream ETC sites. In this condition, uncoupling proteins (UCPs) can decrease proton gradient by leaking protons into the matrix, thereby arresting ROS generation [77]. Moreover, iron-sulfur clusters and reduced FMN of complex I may also act as important sources for ^∙^O_2_^−^ generation. On the other hand, complex III mediates ^∙^O_2_^−^ formation through an electron leakage mechanism arising from autooxidation of ubisemiquinone and reduced cytochrome b [53]. The formation of superoxide may further increase when complex I and complex III are inhibited by rotenone and antimycin, respectively. Inhibition of complex I by rotenone that binds to CoQ_10_ site of the complex can block electron flow from FMN that is fully reduced by high NADH/NAD^+^ ratio, leading to interaction between reduced FMN and O_2_ to form ROS [78]. However, inhibition of the complex by rotenone sometimes shows conflicting results as it can both increase or decrease superoxide formation. For example, increases in superoxide were observed in the human dopaminergic SH-SY5Y cells, mesencephalic neurons, human skin fibroblasts, 3T3-L1 adipocytes, and bovine heart, whereas decreases were found in rat liver mitochondria, mitochondria of rat heart muscle, monocytes and macrophages, and MIN6 cells [79–83]. The exact reason for such discriminating results is unknown. However, it may be possible that substrate-specificity, species- and tissue-specific variation, and surrounding environment (in vivo* or* in vitro) can cause such conflicts. For example, with regard to substrate specificity, rotenone can increase ROS generation in presence of glutamate, whereas it inhibits ROS with succinate [84, 85].

More ROS production occurs when antimycin is used. Because antimycin stabilizes the ubisemiquinone at ubiquinol binding site Q_o_ (outer site) of complex III by preventing electron transfer from Q_o_ → Q_i_ (inner antimycin binding site) → cytochrome c_1_, this in turn causes the ubisemiquinone radical to undergo autooxidation by releasing a singlet electron to be attacked by molecular oxygen leading to ^∙^O_2_^−^ formation [53]. Moreover, myxothiazol can bind to Q_o_ site to prevent electron transfer from QH_2_ at Q_o_ site to Fe-S center, resulting in either increased (probably via reverse electron flow) or decreased (via suppression of mitochondrial inner membrane potential, Δ*ψ*_m_) ^∙^O_2_^−^ formation [86, 87].

On the other hand, ROS generation by complex II should not be underestimated, albeit it is considered to have limited role in ROS release. Complex II appears to produce ROS in a condition of high succinate concentration and membrane potential (Δ*ψ*_m_) when the electrons donated by succinate flow back to complex I via ubiquinone that is associated with increased ROS generation. Complex II can also drive electron flow to complex III at higher succinate level, where leakage of electrons occurs from Q_o_ site of the complex if electron transfer from Q_o_ to Q_i_ is slowed down by antimycin leading to ROS generation [88]. In addition, complex II itself can generate superoxide even at lower concentration of succinate at its flavin site. This is demonstrated by the inhibition of complex II with TTFA that binds to the Q-site of the complex to prevent flavin-mediated ubiquinone reduction. Recently, Anderson et al. showed that TTFA and 3NP (complex II inhibitors) have significantly increased ROS production in comparison to ROS generated by different human skin cells upon exposure to UVA (ultraviolet rays in sunlight), a known ROS stimulator [89]. This supports the notion that complex II inhibitors produce ROS by preventing ubiquinone reduction at Q-site of the complex.

In diabetic milieu, certain factors such as excess reducing equivalents NADH/FADH_2_ [90], increased proton gradient, and membrane potential (Δ*ψ*_m_) [91] reverse electron transport to complex I [92], and increased ATP synthesis resulting from increased electrochemical proton gradient induces mitochondrial ETC to produce ROS. In addition, intracellular glucose homeostasis is impaired in diabetes due to excess uptake of glucose resulting in its increased flux through glycolytic pathway. This causes excessive production of pyruvate and NADH which shuttle into the mitochondrial matrix, where pyruvate is oxidized to produce more NADH and FADH_2_ resulting in excess oxidizing substrates for complex I and complex II. Excessive substrates increase electron donations to ETC, thereby producing high proton gradient, increased membrane potential (reduced negativity in the matrix), and increased ATP synthesis. The excess electron transfer by CoQ_10_ oversaturates complex III where, at a point, electron transport can be blocked resulting in either reverse flow of electron to complex I or electron leakage to O_2_ forming ROS. It is noted that increased ATP synthesis can be stopped by sustained depletion of ADP. This depleted ADP accompanied by attenuated ATP synthesis can eventually lead to ROS production as high electrochemical proton gradient still exists. This observation is substantiated by the study that rat liver mitochondria stimulate ROS generation when incubated with different mitochondrial complex I substrates such as malate, glutamate, and succinate. This stimulated ROS production is attenuated when ADP is added to the incubation medium containing the substrates [93]. Regarding reverse electron flow, Raza et al. demonstrated that electron back flow from complex III/complex IV occurs due to increased substrate-dependent activity of complex I and complex II with decreased activity of complex III and complex IV which facilitates ROS generation. However, inhibition of complex I by rotenone does not necessarily show significant elevation of ROS due to blockade of electron back flow to complex I [94].

### 4.3. Advanced Glycation End Products (AGEs)

AGEs are a group of heterogeneous compounds produced from the nonenzymatic reaction of reducing sugars with the amino groups of proteins, lipids, and nucleic acids. Their generation involves few steps. The first step is “Maillard reaction” which involves the attachment of the carbonyl group (aldehyde or ketone) of reducing sugars with nucleophilic lysine or N-terminal amino groups of a variety of proteins, lipids, and nucleic acids to form Schiff base. In second step, the Schiff bases undergo reorganization to form more stable ketoamines called Amadori products. Amadori products are highly reactive intermediates that include *α*-dicarbonyls or oxoaldehydes. Examples of *α*-dicarbonyls are methylglyoxal, glyoxal, and 3-deoxyglucosone which are also known as precursors of AGEs. In final step, Amadori products undergo further rearrangements through oxidation, dehydration, and degradation to generate highly stable AGEs compounds [95, 96]. AGEs are categorized into 3 classes. These are (1) fluorescent cross-linking AGEs (e.g., pentosidine), (2) nonfluorescent cross-linking AGEs (e.g., imidazolium dilysine cross-links), and (3) non-cross-linking AGEs such as carboxymethyllysine (CML) which arises from the reaction of *α*-dicarbonyls with lysine and arginine [95]. Diabetes increases risk of forming AGEs due to high plasma glucose which plays a primary role in glycation of proteins, lipids, and nucleic acids [97].

AGEs evoke diverse physiological and pathological effects through interaction with their receptors called receptor for AGEs (RAGE). RAGE is multiligand member of immunoglobulin superfamily, usually located on the cell surface of different cells such as macrophages, adipocytes, endothelial cells, vascular endothelial muscle cells, podocytes, and mesangial cells [96, 98, 99]. RAGE comprises an extracellular VC1 ligand-binding domain [97], a single hydrophobic transmembrane domain, and a highly charged -COOH^−^ terminal cytosolic tail that mediates intracellular signaling transduction to evoke diverse cellular effects including altered gene expression [100]. However, AGEs can also induce some receptor-independent pathological effects such as modification of structural integrity of basement membrane by cross-linking with long-lived extracellular matrix (ECM) proteins (e.g., collagen) leading to impaired matrix-matrix or matrix-cell interaction [101].

In addition to many receptor-dependent signaling effects of AGEs, ROS generation resulting from AGE-RAGE-mediated activation of NADPH oxidase and mitochondrial ETC is also prominent. For example, evidence from a study showed that interaction of AGE with its receptor RAGE in INS-1 cells caused early phase of ROS production from mitochondria. Moreover, this AGE-mediated early mitochondrial ROS in turn induce NADPH oxidase to produce more ROS, supporting the notion that AGE-RAGE interaction stimulates ROS generation via mitochondrial or NADPH oxidase pathway [102]. Consistent with this finding, Chuang et al. [103] showed that AGE-RAGE-mediated ROS generation in podocytes had been diminished when podocytes cultured in AGE-enriched medium were transfected with a RAGE-specific siRNA. In another study conducted by Wautier et al., they demonstrated that carboxymethyllysine- (CML-) RAGE-mediated activation of NADPH oxidase in human umbilical vein endothelial cells (HUVECs) significantly increased H_2_O_2_ production, which was diminished upon coincubation of HUVECs with sRAGE and diphenyleneiodonium (DPI), inhibitors of RAGE and NADPH oxidase respectively, suggesting the obvious role of both RAGE and NADPH oxidase in ROS generation [104].

### 4.4. Uncoupled NOS

**Figure 1:**
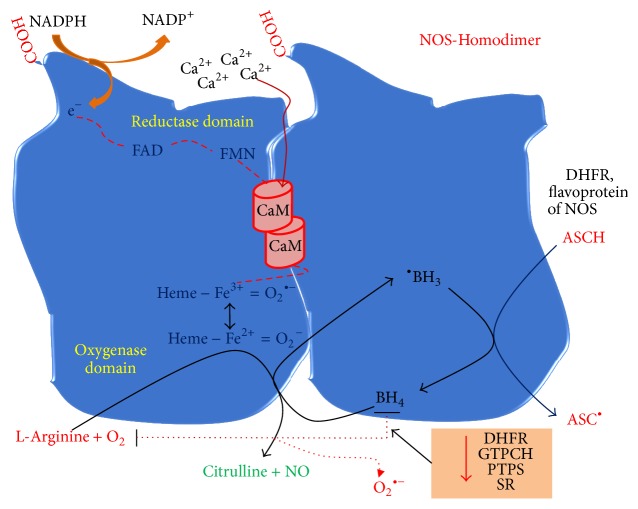
*Pictorial presentation of NO production from arginine by nitric oxide synthase (NOS)*. FAD: flavin adenine dinucleotide; FMN: flavin mononucleotide; NADPH: nicotinamide-adenine-dinucleotide phosphate; CaM: calmodulin; BH_4_: tetrahydrobiopterin; ASCH: ascorbic acid; DHFR: dihydrofolate reductase; GTPCH: GTP cyclohydrolase I; PTPS: 6-pyruvoyl-tetrahydropterin synthase; SR: sepiapterin reductase.

**Figure 2:**
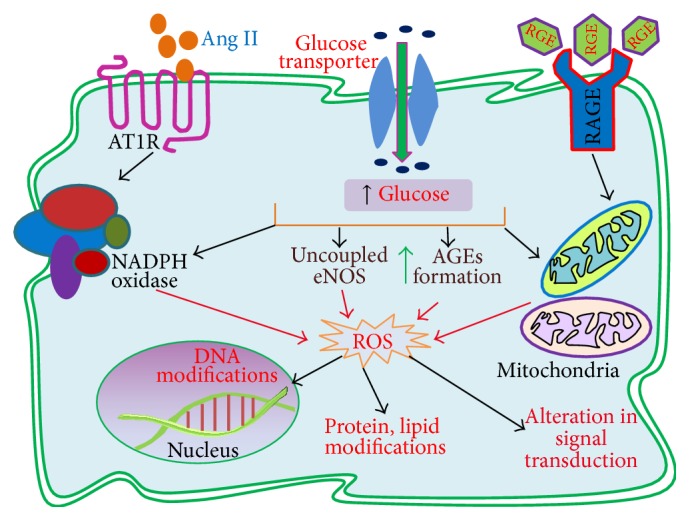
Sources of ROS generation and their impact on cellular components and signaling pathways.

Uncoupling nitric oxide synthase (NOS) is also another important enzymatic source for superoxide generation. Three quite distinct isoforms of NOS have been identified with their different location, regulation, catalytic properties, and inhibitor sensitivity. They are (1) nNOS isoform which is first (also predominantly) found in the neuronal tissue, (2) iNOS isoform which is induced in diverse cells and tissues, and (3) eNOS isoform which is first identified and usually located in vascular endothelial cells. Of these, nNOS and eNOS isoforms are constitutively expressed, whereas iNOS is inducible [105]. Physiologically, all the isoforms are responsible for the production of nitric oxide (NO) or arguably first nitroxyl anion (NO^−^) followed by NO [106], which plays a pivotal role in vasodilation, anti-inflammation, and antiatherosclerosis. Nitric oxide synthase catalyzes the oxidation of its substrate L-arginine in presence of an essential cofactor called tetrahydrobiopterin (BH_4_) to produce NO and L-citrulline. Absence or low levels of arginine and/or BH_4_ can lead to superoxide production instead of NO and this phenomenon is known as NOS uncoupling. Moreover, the catalytic function of NOS also requires the dimerization (e.g., forms homodimers) of the enzyme, impairment of which results in ^∙^O_2_^−^ formation. During NO formation, electron is transferred from C-terminally bound NADPH (in reductase domain) to heme on the N-terminal oxygenase domain of the enzyme (via flavin centers (e.g., FDN and FMN). Reduction of NOS ferric (Fe^3+^) heme by flavin centers enables it to bind with O_2_ and subsequently form ferrous (Fe^2+^)-dioxy species as represented by Fe^3+^ = O_2_^∙−^↔Fe^2+^ = O_2_^−^. This oxygen bound ferrous heme of NOS can preferentially receive one more electron from BH_4_, enabling NOS to hydroxylate L-arginine to initially form N^*ω*^-hydroxyl-l-arginine that is ultimately oxidized to NO and L-citrulline. Any aberration in electron flow within NOS can dissociate ferrous-dioxy complex resulting in generation of O_2_^∙−^ instead of NO from oxygenase domain. Furthermore, in course of catalytic function of the enzyme, BH_4_ is converted to BH_3_^∙^ or dihydrobiopterin (BH_2_) which are no longer capable of reducing heme if they are not either reduced back to BH_4_ or replenished by BH_4_ [105, 107]. It appears that BH_3_^∙^ and BH_2_ can be reduced back to BH_4_ by flavoprotein of NOS or cellular ascorbates [107] and dihydrofolate reductase or dihydropteridine reductase [108], respectively. Additionally, BH_4_ can also be synthesized from GTP by the catalytic action of GTP cyclohydrolase I (GTPCH), 6-pyruvoyl-tetrahydropterin synthase (PTPS), and sepiapterin reductase (SR) to replenish the depleted cellular levels of BH_4_ [109]. Lack of any of these enzymes impairs tetrahydrobiopterin biosynthesis leading to increased superoxide and/or H_2_O_2_ formation by NOS (Figure 1).

All isoforms of NOS can generate superoxide in absence of L-arginine and/or cofactor tetrahydrobiopterin. For example, saphenous veins and internal mammary arteries collected from diabetic patients showed significantly elevated levels of superoxide production especially in the endothelium as demonstrated by fluorescent microtopography. In addition, either denudation of endothelium or inhibition of NOS by N-methyl-l-arginine in diabetic mammary arteries significantly reduced superoxide production suggesting the involvement of eNOS as the mediator of superoxide generation which is reversed in presence of sepiapterin, a BH_4_ precursor [110]. In consistency with this study, Satoh et al. found that glomeruli isolated from streptozotocin-induced diabetic rats increased ROS levels, whereas cofactor BH_4_ and eNOS dimer formation reduced significantly implicating the importance of BH_4_ and eNOS dimerization in the enzyme function. However, addition of BH_4_ or inhibition of eNOS by N^G^-nitro-L-arginine methyl ester (L-NAME) decreased ROS generation significantly, confirming the existence of uncoupled NOS in diabetic glomeruli [55]. On the other hand, inducible NOS has also been reported to produce superoxide which is inhibited in presence of L-arginine as demonstrated by spin trapping of superoxide [111]. Likewise, neuronal NOS purified from brain also increased superoxide levels quantified by spin trapping technique which was also reduced by addition of L-arginine and NOS inhibitor L-NAME [112, 113]. However, data related to iNOS- and nNOS-mediated superoxide production in diabetic condition is still meager. It is notable that superoxide generated by either uncoupled NOS or NADPH oxidase can further react with NO generated by still existing functional NOS, leading to peroxynitrite (ONOO^−^) formation. Peroxynitrite is a potent oxidant which not only damages cellular proteins, lipids, and nucleic acid but also oxidizes BH_4_ resulting in its depletion which in turn leads to NOS uncoupling and dysfunction.

## 5. Fate of ROS

As per above discussion, it is clear that superoxide may produce in both physiological and pathological conditions. Once produced, superoxide is immediately neutralized by cellular superoxide dismutase (SOD) to limit its damaging effects on cellular components. Three isozymes of SOD are found in the cell. They are Cu, Zn-SOD, Mn-SOD, and EC-SOD. Among these, Cu, Zn-SOD is believed to be located in cytosol, whereas Mn-SOD and EC-SOD are thought to be localized in mitochondrial matrix and on the outer surface of cell membranes, respectively [114]. All of these isozymes are capable of dismutating superoxide to form hydrogen peroxide. Hydrogen peroxide is further converted to H_2_O and O_2_ by the action of catalase or glutathione peroxidase (GPx) [52]. Any imbalance between the rates of reactive oxygen species production and their neutralization may lead to accumulation of ROS resulting in oxidative stress. Chronic exposure of tissues to oxidative stress is sufficient to induce a wide range of pathophysiological events leading to eventual cell death.

## 6. Mechanisms of ROS-Mediated Glomerular Renal Injury in Diabetes: Onset, Progression, and End-Stage Damage

Hyperglycemia-induced onset of renal injury is marked by microalbuminuria, glomerular hemodynamic abnormalities, increased kidney and glomerular size, and hyperfiltration. Once these conditions are set in, diverse pathological events are induced due to aberrant signaling cascades with the progress of time. Impaired signaling functions cause a variety of structural and functional changes ranging from increased glomerular basement membrane (GBM) thickness, mesangial expansion, extracellular matrix deposition, glomerulosclerosis, overt proteinuria, and decreased glomerular function and filtration rate to eventual end-stage renal damage. Since diabetic renal injury advances through different stages of structural and functional changes in the glomerulus, we will discuss ROS-mediated renal damage in three steps: onset of injury, progression of injury, and end-stage renal damage.

### 6.1. Onset of Renal Injury through Microalbuminuria

There is an established notion that passage of macromolecules including albumin is highly restricted through normal glomerular capillary wall. However, increased ROS levels in diabetic milieu cause aberration in signaling pathways of different glomerular capillary layers leading to their structural or functional abnormalities which compromise on the glomerular ability of retention of macromolecules. This in turn results in increased leakage of proteins. As all the barriers constitute the capillary wall, it is assumed that each layer's damage by ROS might have a contributory role in protein leakage. Moreover, each layer is likely to communicate with other layer(s) through release of different mediators for the development and the maintenance of functional as well as structural integrity of the glomerular filtration barrier as a composite layer. For example, endothelial layer can communicate with podocytes through secretion of cytokines and growth factors and vice versa [115, 116]. Similarly, podocytes and endothelial cells can also cross-talk through the secretion of various mediators (e.g., type IV collagen) to develop the glomerular basement membrane [117]. This indicates that damage to any of the glomerular layers may induce pathological events to others resulting in excessive fractional clearance of albumin.

Earlier we have discussed microalbuminuria. Here we will focus on how microalbuminuria and hyperfiltration occur at the early phase of renal injury due to ROS-mediated effects inflicted on different glomerular filtration barriers.

#### 6.1.1. ROS-Mediated Damage in Endothelial Layer

From the previous discussion, we have already known that luminal surface of the endothelium is covered by a layer of glycocalyx and endothelial cell coat forming endothelial surface layer (ESL). The glycocalyx is a dynamic hydrated layer largely composed of proteoglycans and glycoproteins of which proteoglycans such as glycosaminoglycans (GAGs) are enriched in heparan sulphate (HS) which gives anionic charge characteristics to the ESL. Interestingly, endothelial glycocalyx can be a major site of action of ROS and different proinflammatory cytokines, which causes degradation of GAGs leading to decreased anionic charges and increased permeability to macromolecules [118, 119]. A study conducted by Singh et al. showed that exposure of glomerular endothelial cell (GEnC) monolayer to ROS such as H_2_O_2_ significantly reduced heparan sulfate (HS) components of GAG and increased albumin passage across GEnC monolayers [120]. The study also found that H_2_O_2_ exposure does not actually inhibit biosynthesis of either total or sulfated GAG chains; rather the exposure causes increased cleavage of HS chain from GAG which was confirmed by quantifying increased levels of HS in GEnC supernatant [120]. In contrary, in vitro culture of GEnC monolayers under high glucose concentration showed decreased biosynthesis of total (both sulfated and nonsulfated) GAG chains with a significant reduction of HS biosynthesis. Moreover, cleavage of HS components from cell-associated GAG was reduced as quantified in GEnC supernatant, which is consistent with the decreased biosynthesis of GAG [121]. Taken together, these observations suggest that GAG, especially its HS chains, is significant for GEnC barrier function and the loss of these components indeed leads to leakage of proteins such as albumin in both high glucose and ROS levels. Though these are in vitro studies that might have some inherent limitations, earlier we have also discussed in vivo studies that have demonstrated similar roles of glomerular endothelial surface layer in preventing free passage of plasma proteins [28, 29]. Besides ROS, other radicals such as reactive nitrogen species (RNS) and carbon centered free radicals can also cause oxidation of core proteoglycan proteins and GAG components such as hyaluronic acid (HA), chondroitin sulfate (CS), and heparan sulfate (HS) leading to their fragmentation and the fragmentation in turn generates more free radicals resulting in aggravated condition of glycocalyx of ESL. In addition, ROS/RNS may also increase the rate of proteolysis of glycocalyx through the activation of matrix metalloproteinases (MMPs) and inhibition of endogenous protease inhibitors [122]. ROS mediated glycocalyx degradation can also be supported by ischemia/reperfusion study, where ROS resulting from ischemia-reperfusion remove endothelial glycocalyx which can be reversed by inhibition of xanthine oxidoreductase, an endogenous ROS producing enzyme bound to HS domains in the glycocalyx [123]. These observations confirm the susceptibility of endothelial glycocalyx layer to diverse radicals including ROS.

Glomerular endothelial cells have also been reported to increase the expression of dysfunctional endothelial nitric oxide synthase (eNOS) due to increased monomeric isoforms instead of dimeric in hyperglycemic condition. Either eNOS impairment or its deficiency results in increased superoxide generation as opposed to NO and the superoxide in turn can scavenge NO decreasing its bioavailability. Attenuation of NO levels impairs endothelium-dependent capillary relaxation as well as vasodilation by enhancing formation of vasoconstrictors and alters renal autoregulation which in combination results in increased glomerular intracapillary pressure and filtration rate (hyperfiltration) which is an early sign of diabetic renal injury [124–126]. Moreover, impaired glomerular endothelial functions along with defective eNOS are involved in many other pathological events that have been discussed later.

#### 6.1.2. ROS-Mediated Damage in Glomerular Basement Membrane

Like endothelium, glomerular basement membrane is also considered to have charge- and size-selective properties because of its anionic heparan sulfate (HS) side chains attached to proteoglycan core proteins (e.g., agrin and perlecan) and extracellular matrix (ECM) network, respectively. It has been found that the heparan sulfate component of GBM can be depolymerized from its core proteoglycan proteins by the action of ROS, whereas uses of ROS scavengers inhibited degradation of HS [127]. However, there is no effect of ROS on proteoglycan core proteins [127, 128], in contrary to other studies which found ROS-mediated inhibition of de novo synthesis of core proteoglycan proteins [129, 130]. The loss of HS from GBM can also be confirmed by using experimental rat model of adriamycin nephropathy in which increased ROS levels are considered to play a role in the disease. Interestingly, this model also showed increased secession of HS from its core proteoglycan proteins, which is a possible effect of ROS [127]. Growing body of evidences showed that the loss of HS components from GBM is the prominent reason for increased permeability of GBM resulting in proteinuria [127–129] except some contradictions [38–40]. Furthermore, HS is thought to interact with other extracellular matrix proteins of GBM including collagen IV and laminin, thereby maintaining the integrity and stability of the basement membrane. Therefore, it is assumed that HS not only confers charge selectivity but also does impart size selectivity indirectly by maintaining ECM networks [127, 131]. In short, it can be said that ROS-mediated damage to HS [127] or proteoglycan core proteins [129] or ECM proteins such as laminin and collagen IV [132] is predominantly involved in increased protein leakage in a variety of human and experimental glomerular disease models.

#### 6.1.3. ROS-Mediated Damage to Podocytes

Podocytes, also known as visceral epithelial cells, are the most restrictive barrier to macromolecules, since podocytes form slit diaphragm with very smaller pores by interdigitating neighboring foot processes that ultimately provide efficient size-based permselectivity. However, its charge selectivity for plasma proteins should not be overlooked as apical membrane domain of podocytes contains anionic surface proteins such as podocalyxin (rich in sialic acid) [133], podoplanin [134], and podoendin [135] which repel against anionic albumin to prevent their easy passage. Therefore, structural and functional integrity of podocytes and its slit diaphragm is critical to maintain restrictive fluid filtration through glomerular filtration barrier. In addition, podocytes can also contribute to the structural and functional development of other glomerular parts including GBM, GEnC, and mesangial cells by establishing a cross-talk with them through secreting various mediators. So, damage to the podocytes not only impairs glomerular permeability but also collapses the whole glomerular architecture leading to advanced renal injury. Damage to the podocytes can be reflected by the reduction of their number which is the balance between podocyte loss and proliferation where increased loss and decreased proliferation result in increased depletion of podocytes.

Hyperglycemia-induced ROS can trigger early loss of podocytes by inducing a variety of pathological events which include apoptosis, detachment of podocytes, foot process effacement, reorganization of cytoskeleton, and dysregulation of any single or a group of podocyte proteins. Due to impaired DNA synthesis and hypertrophy podocyte proliferation may be reduced during cell division [136, 137]. Moreover, cells usually may undergo programed cell death through about a dozen mechanisms that might suggest a broader mechanistic platform for podocyte loss. Some of these mechanisms may include autophagy (starvation-induced cell death), aberrant cell cycle progression (e.g., mitotic catastrophe), abnormal proliferation, anoikis (cell death due to absence of cell-matrix interactions), antosis (cell-in-cell death, cannibalism), and necrosis (upregulated lysis of the cell membrane) [138]. Though extensive discussion on all mechanisms is beyond the scope of our current review, we will briefly discuss apoptosis, detachment of podocytes, foot process effacement, autophagy, and cell cycle abnormalities as potential mechanisms of podocyte death and loss.

*(1) Apoptosis.* Podocytes can be a target of ROS-mediated damage, since many ROS generating pathways are activated in podocytes in high glucose ambience. Several studies have reported that multicomponent complexes of NADPH oxidase [139, 140], mitochondrial respiratory chain [141], and AGEs [142] are the major sources of ROS in podocytes. Moreover, NADPH oxidase [136, 143, 144] and mitochondrial ETC [136] are reported to be activated in podocytes cultured in high glucose, resulting in increased ROS production.

**Figure 3:**
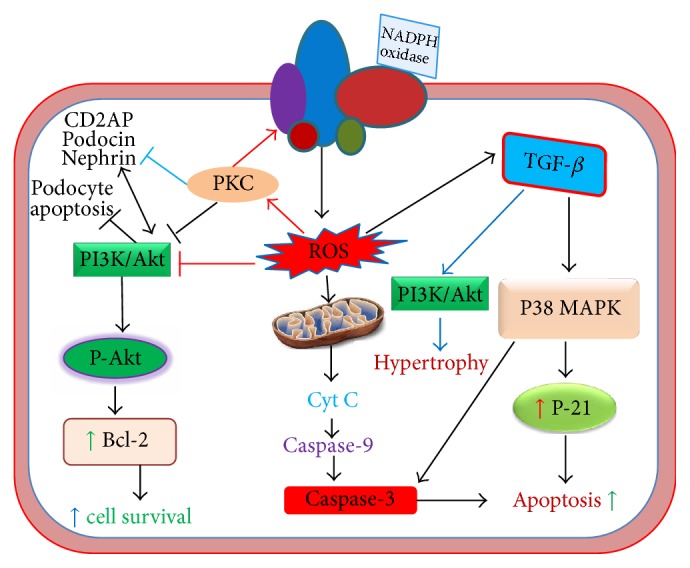
Major signaling pathways for induction of apoptosis and hypertrophy of podocyte and mesangial cells.

Reactive oxygen species induce dysregulation of different redox signaling cascades in the podocytes causing their apoptosis or detachment. In doing so, high glucose or ROS can upregulate and activate diverse proinflammatory cytokines and transcription factors, proapoptotic molecules, and growth factors. Recently, using type 1 and type 2 diabetic models of mice, Susztak et al. [136] demonstrated that ROS generated from NADPH oxidase and mitochondrial pathways have significantly increased apoptosis of podocytes with the onset of diabetes through increased activation of proapoptotic mediator p38-MAPK (p38-Mitogen activated protein kinase) and caspase-3. The podocyte apoptosis precedes its depletion which leads to increased urinary albumin excretion. p38-MAPK and caspase-3 are downstream proapoptotic mediators that are required by TGF-*β* which is highly expressed and activated in podocytes, resulting in their increased apoptosis [145]. However, SMAD7 can independently induce podocyte apoptosis without requiring any of p38-MAPK and caspase-3 or TGF-*β*. Moreover, TGF-*β* can enhance synthesis of SMAD7 that can amplify TGF-*β*-induced p38-MAPK and caspase-dependent apoptosis. TGF-*β* can also increase Bcl2-associated X protein (Bax) expression through induction of Bax gene transcription and mitochondrial translocation of Bax protein that results in cytochrome c release from mitochondria and subsequent activation of caspase-3 (Figure 3) [146]. In consistency with these findings, Lee et al. reported that both Bax and activated caspase-3 have been significantly overexpressed in the glomeruli isolated from diabetic rats and podocytes cultured in high glucose levels with resultant apoptosis [147]. Interestingly, both high glucose and ROS levels can increasingly induce TGF-*β* expression in various tissues including the glomerulus [148–150]. Once TGF-*β* is upregulated, it can further enhance ROS generation via activation of NADPH oxidase complexes [151] and mitochondrial respiratory function [152] leading to exacerbation of TGF-*β*-induced apoptosis and detachment of podocytes. In addition to induction of podocyte apoptosis and detachment, TGF-*β* indeed activates diverse signal transduction pathways to elicit pathological changes to the architecture and function of the glomerulus which has been discussed in greater detail later.

*(2) Detachment.* Podocyte detachment is also promoted by ROS through activation of different signaling pathways. Podocytes are attached to the GBM via cell surface adhesion proteins such as *α*3*β*1 integrin and dystroglycans (DGs). Impaired interaction with GBM or decreased synthesis of these proteins can apparently lead to podocyte detachment. Accumulating evidences show that high glucose and ROS can downregulate the expression of *α*3*β*1 integrin, an important podocyte anchoring receptor [153–155]. Decreased expression of *α*3*β*1 integrin can lead to increased podocyte detachment due to loss of FPs, resulting in enhanced proteinuria. This evidence is supported by a study where deletion of podocyte-specific integrin *α*3 subunit in mice caused massive proteinuria before 3 weeks and nephrotic syndrome by 6 weeks of their age [156]. Detachment of podocytes is substantiated by their presence in the urine in experimental and clinical studies of both diabetic and nondiabetic glomerular diseases. Many of these urinary podocytes are even viable and accompany micro to overt proteinuria and can be recognized as another important marker for glomerular disease [155, 157, 158].

*(3) Foot Process Effacement*. Foot process effacement (FPE) is characterized by retraction of the foot processes resulting in shortening of its length and increasing the width and the widening of foot processes are associated with the reduction in the podocytes number. The FPE usually replaces slit diaphragm by occluding junctions leading to sealing of the filtration slits. Reportedly, FPE is induced by reorganization of cytoskeletal proteins (e.g., *α*-actinin-4 and synaptopodin), dysregulation of slit diaphragm proteins, and interference with podocyte-GBM interaction which increasingly result from oxidative stress-induced injury in diabetic settings. It has been observed that deletion or mutation of any of the slit diaphragm-associated proteins such as nephrin, podocin, P-cadherin, CD2AP, and zonula occludens-1 (ZO-1) accelerates foot process effacement followed by proteinuria [137, 159]. Attenuated expression and/or increased loss of these slit proteins have also been observed in ROS-mediated diabetic and nondiabetic experimental models of glomerular abnormalities. Very recently, do Nascimento et al. [160] assessed mRNA levels of various podocyte proteins in urine collected from diabetic, prediabetic, and control patients and observed that mRNA levels of slit diaphragm proteins (e.g., nephrin and podocin) and podocyte cytoskeletal proteins (e.g., *α*-actinin-4 and synaptopodin) have been significantly increased in diabetic patients with normoalbuminuria, microalbuminuria, and macroalbuminuria. Increased urinary expression of these proteins in normoalbuminuric diabetic subjects suggests that podocyte damage may occur in early stage of diabetic injury. Similarly, nephrin expression has been inversely decreased with regard to ROS levels in mouse podocytes cultured in high glucose compared to normal glucose treatment group. Similar result was also found in OLETF diabetic rat models. Treatment with taurine and resveratrol (antioxidant agents) has restored nephrin mRNA levels and improved albuminuria, indicating the role of ROS in downregulation of nephrin in diabetes [161]. Furthermore, streptozotocin-induced diabetic spontaneously hypertensive rats showed decreased nephrin expression with consequent albuminuria which may result from reactive oxidants [162].

On the other hand, in nondiabetic in vivo and in vitro studies treated with puromycin aminonucleoside (PAN), loss of nephrin and podocin expression has been observed in line with increased foot process effacement and cytoskeletal actin reorganization of podocytes. Actin reorganization that is accompanied by loss of synaptopodin may induce FPE. These pathological modulations are found to be caused by an underlying mechanism of ROS generation and subsequent activation of p38-MAPK pathway. Triptolide has showed restoration of nephrin and podocin levels with remarkable improvement in cytoskeleton and foot processes by reducing ROS levels and p38-MAPK activation and ultimately decreased proteinuria [163]. In consistency with these findings, another recent study conducted by Lan et al. [164] demonstrated that slit diaphragm constituting proteins such as nephrin, podocin, and CD2AP and cytoskeletal synaptopodin are decreased in morphine treated mice with increased foot process retraction and cytoskeleton disruption. This can be attributed in part to morphine-induced oxidative stress which is likely to activate JNK, AKT, and p38 pathways. However, downregulation of nephrin, podocin, and CD2AP by activated AKT in morphine treated mice is a contradiction to the evidence that nephrin, podocin, and CD2AP themselves activate AKT via activation of PI3K to promote survival of podocytes [165]. It is pertinent to note that PI3K/AKT signaling can contribute to hypertrophy of mesangial cells upon activation by TGF-*β*1 [166], whereas podocytes may also undergo hypertrophic change in response to high glucose, intraglomerular pressure, and Ang II (Figure 3) [167]. Although ROS can activate PI3K/AKT signaling pathway by inducing AKT phosphorylation [54, 168], they can also be accountable for inactivation of AKT by its diminished phosphorylation [103, 169, 170]. For example, AGE-induced ROS generation as evident in diabetic milieu is likely to inhibit phosphorylation of AKT, leading to its deactivation which in turn promotes podocyte apoptosis [103]. In addition, ROS generated from mitochondrial dysfunction of aldosterone-treated podocytes (both in vitro and in vivo) are responsible for significant reduction of nephrin [141], which impairs nephrin-AKT interaction, resulting in podocyte death (Figure 3).

Moreover, diabetes is characterized not only by oxidative stress levels but also by other complications including insulin resistance, reduced adiponectin, and increased inflammatory mediators which are highly common in obese subjects. Thus, obese patients with diabetes are more susceptible to renal injury. This notion is supported by a recent study where the investigators using normoglycemic Zucker-fatty rats showed that some podocyte proteins such as nephrin, podocin, podocalyxin, and CD2AP are remarkably downregulated which can be attributed to increased oxidative and inflammatory mediators [171]. In conformation with this, Sharma et al. [172] demonstrated that obese patients with normoglycemia showed negative correlation of adiponectin with albuminuria. This indicates the importance of adiponectin in regulation of podocyte permeability by integration of SD-associated proteins. This is further supported by the findings that zona occludens-1 (ZO-1), an SD protein, can be translocated from cytoplasmic site to the plasma membrane via an AMPK-dependent pathway. Interestingly, ROS generating Nox4 can downregulate adiponectin and AMPK levels leading to impaired translocation of ZO-1 which cause increased permeability of slit diaphragm followed by excessive albuminuria. Similar observations were also found in adiponectin knock-out mice with or without diabetes suggesting that adiponectin-mediated regulation of SD permeability is critical to prevent albuminuria.

All the hyperglycemia-induced ROS-mediated pathological events discussed above are adequate to initiate protein leakage across structurally and functionally impaired glomerular barrier resulting in primary urine with excessive proteins. Though increased leakage of protein through glomerulus is the initiating point for microalbuminuria, other factors such as inability of renal tubule to increase protein reabsorption corresponding to increased protein levels in the tubular filtrate and decreased capacity of tubular reabsorption of proteins due to increased injury to the tubular cells can be held responsible for eventual increased urinary excretion of protein or albumin leading to microalbuminuria which, with progress of time, results in macroalbuminuria with advanced pathological changes in the kidney.

*(4) Autophagy in Podocytes.* Autophagy is an evolutionary conserved housekeeping process by which eukaryotic cells themselves degrade and recycle their cytoplasmic macromolecules and organelles in defense against cellular stress. This cellular self-degradation pathway is activated under environmental stress conditions and various pathological situations and is important for cell survival during these stressful conditions. Based on morphological and mechanistic characteristics, three forms of autophagy are recognized to date: macroautophagy, microautophagy, and chaperone-mediated autophagy (CMA) [138, 173]. Macroautophagy involves sequestration of any type of cellular contents including large organelles such as mitochondria and ribosomes within a double membrane bound vacuole called the autophagosome. In the second form of autophagy, microautophagy, cytosolic macromolecules and small organelles are directly engulfed by the lytic organelles through invagination of the lysosomal or vacuolar membrane. Chaperone-mediated autophagy is quite distinct from other types of autophagy and involves elimination of no organelles. This mechanism is selective for digestion of proteins that contain a certain amino acid sequence, namely, KFERQ (for lysine-phenylalanine-glutamate-arginine-glutamine). It has been noted that impaired CMA increases macroautophagy, implying an interaction between different forms of autophagy [173].

Podocytes are terminally differentiated cells with a limited proliferative capacity. Therefore, the fate of a podocyte depends on its ability to cope with the stress. Fortunately, podocytes exhibit a high level of autophagy even under nonstress conditions, suggesting that podocytes need to keep cellular homeostasis under basal conditions [174].

Evidently, autophagy plays an important renoprotective role by mainly maintaining homeostasis of podocytes in diabetic nephropathy. It has been manifested by podocyte-specific expression of autophagy related proteins such as Beclin-1, Atg5–Atg12, and LC3 (rat microtubule-associated protein 1 light chain 3) which results in increased basal level of autophagy in podocytes [175]. However, under certain diabetic conditions, including high glucose in vitro conditions, high basal levels of autophagy in podocytes became defective and defective autophagy facilitates podocyte injury. This evidence is supported by decreased expression of Beclin-1, Atg5–Atg12, and LC3 both in podocytes of STZ-induced diabetic mice and in cells cultured in high glucose [175]. In agreement with this observation, a very recent study showed insufficient autophagy in podocytes of diabetic patients and rodents with massive proteinuria which indicates autophagy to be implicated in the pathogenesis of diabetic nephropathy [176].

The mechanism underlying diabetes-induced impairment of podocyte autophagy is still ambiguous. However, in podocytes of diabetic mice and patients, mTORC1 (mammalian target of rapamycin complex 1) is highly activated and may be involved in the mechanisms of diabetes-induced autophagy inhibition in podocytes [177]. Interestingly, increased mTOR activity accompanied by human diabetic nephropathy induces early glomerular hypertrophy and hyperfiltration, whereas genetic deletion of mTORC1 in mouse podocytes results in proteinuria and progressive glomerulosclerosis, suggesting the requirement for tightly balanced mTOR activity in podocyte homeostasis [177]. Moreover, podocyte-specific activation of mTORC1 results in many features of DN, such as mesangial expansion, glomerular basement membrane (GBM) thickening, podocyte loss, and proteinuria in nondiabetic mice. Since mTORC1 attenuates autophagy, inhibition of mTORC1 can restore the autophagy of podocytes to basal levels resulting in the improvement of the features of diabetic nephropathy [178]. This has been supported by evidence that treatment of experimental models of type 1 and type 2 diabetic animals with the mTORC1 inhibitor, rapamycin, reduced the development of diabetic nephropathy [179].

*Cell Cycle Abnormalities in Podocytes.* The cell cycle involves five tightly controlled phases, that is, G_0_ (resting) phase, G_1_ phase, S phase, G_2_ phase, and M (mitosis) phase. Proper cell cycle progression through all these phases can give rise to new cells which is very important for cellular homeostasis in tissue. Cell cycle entry begins with G_1_ phase and ends within G_0_ phase where newly divided cells remain quiescent and fulfill their physiological functions with the tissue. Mature podocytes are thought to be quiescent cells arrested in G_0_ (resting) phase. The cell cycle has also some integrated checkpoints to ensure the fidelity of the cell division. For example, the first checkpoint, G_1_/S, checks for the presence of damage DNA, and if any damaged DNA is found, it stalls for DNA repair. The G_2_/M checkpoint will determine whether or not the cell proceeds to complete mitosis. Finally, metaphase or spindle checkpoints ensure proper chromosome alignment prior to cell division. In addition, normal cell cycle functions are regulated by three classes of proteins: cyclic proteins (cyclins), cyclin-dependent kinases (CDKs), and cyclin-dependent kinase inhibitors (CKIs). Podocytes express cyclin A, B1, and D1 as well as CDK inhibitors, such as p21, p27, and p57. Any abnormality in cell cycle components and/or checkpoints which is beyond the scope of automatic repair (e.g., DNA damage) may warrant for cell cycle arrest at distinct restriction points mediated by p53 and p27 cell cycle regulatory proteins [138, 180].

Mature podocytes reduce expression of Ki-67, a proliferation marker, cyclin A, and cyclin B1, while CKIs and cyclin D1 are intensively increased. Cyclins and CDKs can be modulated in human and experimental podocyte injury. For example, in the cellular type of human FSGS (focal segmental glomerulosclerosis), studies have found absent p27, p57, and cyclin D1 expression and increased cyclin E, cyclin A, cyclin B1, CDK2, and p21 [138]. In adriamycin-induced podocyte injury, the presence of CDK inhibitor p21 is protective for podocytes in this model of toxic podocytopathy. Conversely, in membranous nephropathy, podocytes upon immune-mediated injury increase DNA synthesis in S phase and upregulation of cyclin A and CDK2 and finally enter mitosis but are unable to divide resulting in multinucleated podocytes [180].

Podocyte hypertrophy is a characteristic of diabetic nephropathy. It occurs in different diabetic animal models due to increased expression of CKIs. For example, Zucker diabetic rats and db/db mice, both models of type 2 diabetes, or type 1 models, induced by streptozotocin administration, increase the expression of p27 and p21 resulting in podocyte's cell cycle arrest in response to injury induced DNA damage and this in turn causes glomerular hypertrophy and development of progressive renal failure [180, 181]. Interestingly, exposure of cultured mouse podocytes to cyclic mechanical stretch showed decrease in cyclins D1, A, and B1 and increase in CDK inhibitors p21 and p27, prompting the podocyte to adopt a hypertrophic phenotype [181]. Similarly, AGEs which are abundantly produced in hyperglycemic milieu can induce podocyte hypertrophy through upregulation of CDK inhibitor p27, which causes cell cycle arrest [57]. All these cell cycle related abnormalities are prominent during the early stages of diabetic nephropathy which may progress toward irreversible damage through changes of podocytes from their hypertrophic to increased apoptotic phenotypes.

### 6.2. Glomerular Hyperfiltration

Increased glomerular filtration rate (GFR) or hyperfiltration also marks the early sign of diabetic renal injury and may play a major role in the pathogenesis of diabetic nephropathy. Glomerular hyperfiltration occurs due to increased dilation of afferent arterioles leading to increased blood flow to the glomeruli. This afferent arteriolar dilation can be attributed to increased prostaglandin E2 synthesis, impaired responsiveness to vasoconstrictors (i.e., thromboxane and norepinephrine), elevated levels of atrial natriuretic peptide (ANP), and hyperglycemia-mediated inactivation of tubuloglomerular feedback (TGF) [182]. In diabetes, inactivated TGF results from increased glucose reabsorption along with Na^+^ from the proximal tubule leading to decreased sodium delivery to macula densa (MD) cells. This phenomenon can further be interpreted by the fact that hyperglycemia usually increases glucose concentration in tubular filtrate and upregulates expression of both sodium glucose linked transporters-1 and -2 (SGLT1 and SGLT2) in the proximal tubule that causes increased cotransportation of glucose and Na^+^ [182, 183]. However, role of TGF in hyperfiltration in diabetes has been debated since A1 adenosine-receptor (AA1R) null mice, previously shown to lack a functional TGF, still exhibit pronounced hyperfiltration when diabetes is induced [183, 184]. In addition, diabetic hyperfiltration may also result from increased pressure gradient across glomerular membrane which arises from increased capillary hydrostatic/colloidal pressure and reduced hydrostatic pressure in Bowman's capsule or proximal tubule. Interestingly, pressure in the proximal tubule is reduced due to increased reabsorption of Na^+^ and Cl^−^ resulting from persistent hyperglycemia-mediated oxidative stress [183]. Additionally, prostaglandin E_2_ (PGE_2_) mediated reduction of hyperfiltration was explained by Kiritoshi et al. who showed increased PGE_2_ synthesis in human mesangial cells (HMCs). They also found that prostaglandins synthesis in HMCs is increased due to ROS-mediated upregulation of cyclooxygenase-2 (COX-2) mRNA and increased activation of NF-*κ*B. Prostaglandins in turn may modulate afferent arteriolar vasoconstriction after stimulation of TGF [185]. Moreover, high glomerular capillary pressure elicited from increased vasoconstriction of efferent arterioles also may contribute to hyperfiltration [186].

## 7. Progression of Renal Injury through Diverse Signaling Pathways

Though microalbuminuria may be initiating step for glomerular damage, progression of damage actually is achieved through activation of diverse pathological pathways. We have already discussed some of the signaling molecules that evoke some structural and functional damage to the filtration barrier to increase glomerular permeability. Now we will have a holistic view on some more signaling mediators in greater detail which are responsible for advanced pathological damage to the glomerulus if initial symptoms are not corrected. Of note, signaling mediators can be activated in any part of the glomerulus in response to high glucose, AGEs, and/or ROS. However, their activation in any glomerular cell type may affect surrounding cells as the whole glomerulus acts as a coordinated unit to regulate its functions.

### 7.1. Protein Kinase C (PKC)

PKC is also an important signaling molecule playing central role in glomerular injury. In high glucose ambience, PKC is activated by diacylglycerol (DAG), a signaling molecule increasingly produced from an intermediate product of glycolytic pathway such as glyceraldehyde-3-phosphate (G3P) which is abundantly produced from high glucose flux into glycolytic pathway. Interestingly, under high glucose conditions, PKC can also be activated by higher concentrations of ROS, perhaps through tyrosine phosphorylation or DAG synthesis. Moreover, PKC-*β*2 can stimulate NADPH oxidase to produce more ROS resulting in vicious cycle of enhanced PKC activation (Figure 3) [187–189]. Activation of PKC (e.g., PKC-*α*) causes proteinuria by degradation of nephrin of slit diaphragm. Activated PKC can also attenuate expression of P-cadherin mRNA and protein in experimental glomeruli and high glucose-stimulated podocytes, which suggests a potential role of P-cadherin loss in the development of excessive proteinuria [187, 190]. In addition, the activated PKC can promote endothelial dysfunction and increased production of endothelin-1, TGF-*β*, VEGF, and NF-*κ*B leading to alteration in blood flow, capillary permeability, and extracellular matrix deposition.

### 7.2. Transcription Factors

**Figure 4:**
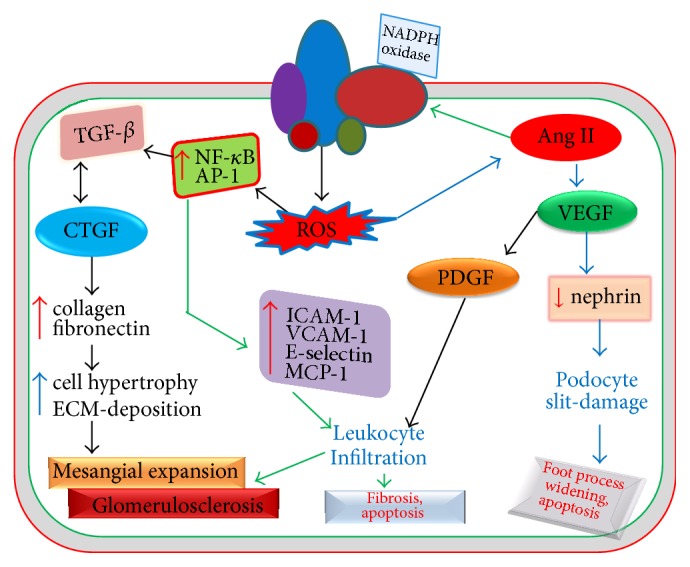
Major signaling pathways for induction of ECM accumulation following mesangial expansion, increased GBM, glomerulosclerosis, and fibrosis. This results in subsequent end-stage renal damage.

*Nuclear Factor-Kappa B (NF-κB).* This is a redox-sensitive transcription factor that can be activated by a wide variety of stimuli including oxidative stress in various renal cells such as podocytes and endothelial, mesangial, and tubular cells [191]. ROS-mediated activation of NF-*κ*B can interfere with the transcription of a wide range of proinflammatory and profibrotic genes coding for cytokines, adhesion molecules, and growth factors causing vascular dysfunction, atherosclerosis, and inflammation. Therefore, proinflammatory cytokines such as TNF-*α*, IL-1*β*, IL-2, IL-6, and IL-12, leukocyte adhesion molecules (e.g., E-selectin, VCAM-1, and ICAM-1), growth factors (TGF-*β*), and chemokines (MCP-1) are upregulated during persistent oxidative stress-induced NF-*κ*B activation (Figure 4) [192]. In resting cells, NF-*κ*B is continuously present in inactive state, when NF-*κ*B remains bound to the inhibitory I*κ*B proteins, preventing its translocation to nucleus. Activation of NF-*κ*B requires the phosphorylation of I*κ*B which causes ubiquitination of I*κ*B implying its destruction by proteasome. I*κ*B kinases (IKK) can phosphorylate I*κ*B to facilitate ubiquitination and degradation of I*κ*B followed by release of I*κ*B-bound NF-*κ*B, thereby translocating NF-*κ*B to the nucleus to initiate gene transcription [191]. However, ROS have also been considered to phosphorylate I*κ*B on its tyrosine residue instead of serine; the latter is usually seen in IKK-mediated phosphorylation, thus translocating NF-*κ*B to the nucleus. Besides ROS-mediated activation, however, ROS have also been reported to inhibit NF-*κ*B binding with DNA by oxidizing its Rel homology domain in nuclear region showing differential roles in cytoplasm and nucleus. These differences can be attributed to the study of different upstream pathways and cell-specific differences [193].

### 7.3. Activator Protein-1 (AP-1)

AP-1 is another redox-regulated transcription factor involved in transcription of various inflammatory genes in response to activation by diverse stimuli. ROS can activate AP-1 through phosphorylation of upstream MAPKs such as ERK and p38 kinases as shown by a study [194]. In another study, it was shown that high glucose can lead to PKC*β*1-mediated ROS generation through NADPH oxidase with subsequent RhoA activation in mesangial cells. This RhoA in turn activates downstream AP-1 through Rho kinase leading to activation of TGF-*β*1 [195]. In consistency with these observations, Weigert et al. [196] demonstrated that AP-1 activation is responsible for increased TGF-*β*1 expression through PKC- and p38-MAPK-dependent pathways.

### 7.4. Hypoxia Inducible Factor (HIF)

HIF is a heterodimeric transcription factor that is composed of two subunits, an oxygen sensitive HIF-*α* subunit and a constitutively expressed HIF-*β* subunit. HIF-1*α* was the first isoform of HIF-*α* to be cloned. HIF-1*α* is activated in response to cellular hypoxia and induces transcription of various genes encoding erythropoietin, VEGF, glucose transporters, CTGF, and PAI-1, all of which are considered to aggravate extracellular matrix deposition in the glomerulus. Hypoxia, a common inducer of HIF-1*α*, can occur in the diabetic kidney resulting from increased consumption of oxygen by renal tubule and superoxide-mediated increased Na-K-2Cl cotransporter activity in the thick ascending limb (TAL) [197, 198]. In high glucose condition, HIF-1*α* has been upregulated in mesangial cells as evidenced in streptozotocin-induced diabetic mice and in vitro studies. Moreover, in addition to hypoxia, other factors such as angiotensin II, TGF-*β*1, PKC, and ROS which are found to be upregulated in diabetes can also activate HIF-1*α*, thereby exacerbating glomerular injury even in nonhypoxic condition [9]. For example, a study reported that Ang II increased HIF-1*α* protein levels in mesangial cells through stimulation of ROS generation which in turn activate PI3K/Akt pathway [199]. Since HIF-1*α* is capable of increasing transcription of profibrotic genes, it can greatly contribute to the renal fibrosis in diabetes with or without hypoxia leading to end-stage renal failure [200].

Other transcription factors including CREB (c-AMP-response-element-binding protein), NFAT (nuclear factor of activated T cells), and Sp1 (stimulating protein 1) are also activated in hyperglycemic milieu. These transcription factors can also regulate genes related to inflammation and ECM turnover [201]. Ang II-mediated podocyte injury can be induced by CREB which carries signal from calmodulin-dependent protein kinase II (CaMK II) to downstream Wnt/*β*-catenin signaling pathway to increase Wnt mRNA expression and *β*-catenin phosphorylation leading to inhibition of podocin and nephrin expression. Inhibition of CREB has improved podocyte injury by restoring podocin and nephrin levels confirming its role in renal injury [202].

### 7.5. Inflammatory Cytokines

Cytokines are small, nonstructural proteins with low molecular weights having autocrine, paracrine, and juxtacrine effects and very complex activities. They can act as regulators of host response to infection, immune response, trauma, and inflammation with their both pro- and anti-inflammatory role based on the type of cell, the time of action, and cellular environment. There are a lot of proinflammatory cytokines that can be activated in response to high glucose or oxidative stress. These include IL-1, IL-6, IL-18, and TNF-*α* which are mainly involved in the development and progression of diabetic nephropathy [203]. These cytokines are also transcribed by various transcription factors in inflammatory conditions.

IL-1*β* expression is increased in the glomeruli of streptozotocin-induced diabetic rat model which has been accompanied by augmentation in chemokines (MCP-1) and cell adhesion molecules (VCAM-1 and ICAM-1) expression. Macrophage infiltration can accompany MCP-1, VCAM-1, and ICAM-1 overexpression in different renal cells, including endothelial, mesangial, and tubular epithelial cells and also concomitantly overexpress *α*1-chain type IV collagen. This effects cause structural changes in renal cells by accumulating ECM proteins resulting in glomerulosclerosis in both type 1 and type 2 diabetic models [204, 205].

IL-6 has also been reported to be significantly high in type 2 diabetic patients with nephropathy (DN) in comparison to DM patients without DN. Analysis of kidney biopsies in patients with type 2 DN evidenced increased expression of IL-6 in cells infiltrating mesangium, interstitium, and tubules. Moreover, there is a positive relationship between mesangial expansion (glomerulopathy) and expression of IL-6 mRNA in both mesangial cells and podocytes, implying an important role of IL-6 in influencing extracellular matrix dynamics at mesangial and podocyte levels [206]. Recently, Choudhary and Ahlawat analyzed serum levels of IL-6 in type 2 diabetic patients to find a correlation between IL-6 and albuminuria. They have found significant positive correlation between IL-6 and albuminuria, suggesting contributory role of IL-6 in renal injury [207]. In conformation with this observation, another study also found positive correlation between IL-6 and UAE in type 1 DM patients [208]. These observations implicate IL-6 as having pathological role in diabetic renal injury progression toward renal failure.

IL-18 is potent inflammatory cytokine that is involved in different functions, such as induction of interferon-*γ* (IFN-*γ*) [209], production of proinflammatory cytokines (IL-1 and TNF-*α*) [210], overexpression of ICAM and VCAM molecules [210, 211], and increased apoptosis of endothelial cells via TNF-*α* and Fas (members of TNF family) [212]. IFN-*γ* further stimulates functional chemokine receptors expression in human mesangial cells [213]. Interestingly, similar to IL-6, IL-18 has also been reported to be enhanced in serum and urine samples of patients with diabetic nephropathy compared to controlled subjects, and higher IL-18 levels in turn increase UAE as evidenced by many clinical studies [214–216]. Therefore, it is evident that elevated IL-18 in diabetes is a predisposing threat to the renal dysfunction [216].

TNF-*α* is another cytokine having pronounced proinflammatory roles and mainly produced in monocytes, macrophages, and T cells. However, host renal cells such as mesangial, glomerular, endothelial, and tubular cells also produce TNF-*α* [217–220]. TNF-*α* mediates cellular effects via two receptors: (1) TNF-*α* receptor-1 (TNFR1) and (2) TNF-*α* receptor-2 (TNFR2). TNFR1 modulates immune response through IL-6 synthesis and apoptosis through apoptotic signal-regulating kinase-1 (ASK-1) and NF-*κ*B, whereas TNFR2 mediates proinflammatory effects in glomerulonephritis [205]. Prominent actions of TNF-*α* on renal cells are the activation of second messenger systems, transcription factors, synthesis of growth factors, receptors, cytokines, cell adhesion molecules, and more importantly promotion of local ROS generation in diverse cells, including mesangial cells [206, 221]. TNF-*α* can also induce changes of intraglomerular blood flow and GFR resulting from hemodynamic imbalances between vasoconstrictors and vasodilators [222] and alters endothelial permeability. In addition, it can alter location of receptors involved in cell-cell adhesion and prevents the formation of F-actin stress fibers leading to modification of intercellular junctions followed by loss of endothelial permeability [223]. TNF-*α* can also induce cytotoxicity and apoptosis [224, 225]. Many experimental diabetic rat models showed increased TNF-*α* levels in renal cortex [226, 227], whereas clinical data of type 2 diabetic patients exhibited higher serum levels of TNF-*α* with significant microalbuminuria [214].

### 7.6. Other Cytokines/Growth Factors (GFs)

Growth factors are activated by different effectors to induce secretion of matrix forming proteins to increase mesangial expansion as well as GBM thickness and express many cellular entities to promote cellular hypertrophy, apoptosis, and foot process effacement. Major GFs that play critical role in the pathogenesis of renal injury include TGF-*β*, VEGF, CTGF, and PDGF.

#### 7.6.1. Transforming Growth Factor-*β* (TGF-*β*)

TGF-*β* is a widely studied multifunctional cytokine that modulates cell proliferation, differentiation, apoptosis, adhesion, and migration of diverse cell types and induces the production of ECM proteins. TGF-*β* is expressed in many cell types including immature hematopoietic cells, activated T and B cells, macrophages, neutrophils, and dendritic cells which are sensitive to its effects [145]. It induces podocyte apoptosis via different downstream effectors including p38-MAPK, Smad, Bax, and caspase 3 (discussed above). Moreover, podocyte apoptosis can also be induced through TGF-*β*-mediated p21, a cyclin-dependent kinase (CDK) inhibitor [228]. This idea is supported by the findings that, like TGF-*β*, p21 has been reported to be increased in different experimental models of glomerular diseases such as membranous nephropathy (PHN model) [229], streptozotocin-induced diabetic nephropathy [230], and minimal change nephropathy. Wada et al. [228] reported that TGF-*β* increases p21 levels in cultured podocytes which coincides with their increased apoptosis. This is confirmed by the findings that TGF-*β* treatment of p21-null podocytes in culture decreased apoptosis, whereas wild type enhanced apoptotic response. However, transfection of p21 in p21-null podocytes has retained the apoptotic response to TGF-*β*, suggesting the implication of p21 as a downstream effector in TGF-*β*-induced apoptosis. Moreover, TGF-*β* can also induce apoptosis mesangial and glomerular endothelial cells. In addition, p21 and its another family member, p27, can also induce hypertrophy of mesangial cells as well as podocytes by inhibiting cell cycle progression [138, 230].

In addition to its apoptotic role, TGF-*β* can stimulate MCs to induce ECM deposition by producing types I, III, and IV collagen, laminin, and fibronectin and by inhibiting matrix degrading proteins called MMPs. Matrix expansion results in mesangial cell hypertrophy and apoptosis and decreases glomerular surface area for fluid filtration which leads to gradual diminution of GFR. In addition, TGF-*β* can also promote similar ECM-producing proteins in GBM, thereby increasing its thickness [231, 232]. Interestingly, high glucose, AGEs, and ROS push the disease forward to more complications by inducing TGF-*β* and other matrices and apoptosis influencing effector molecules. Increased mesangial expansion and GBM thickness ultimately cause more podocyte apoptosis advancing the disease toward renal failure.

#### 7.6.2. Vascular Endothelial Growth Factor (VEGF)

VEGF being expressed predominantly by podocytes and in some cases by mesangial cells in the kidney can induce angiogenesis and vascular permeability. VEGF elicits its action by interacting with its receptor located on the endothelium and mesangial cells [233, 234]. Many experimental diabetic rat models, such as type 1 (STZ-induced diabetic rats) and type 2 (e.g., Otsuka-Long-Evans-Tokushima-Fatty (OLETF) rats and Zucker Diabetic Fatty (ZDF-rats)) rats, demonstrated elevated expression of VEGF mRNA in the glomerulus [235–237]. In in vitro study, podocytes cultured in high glucose increases VEGF mRNA expression by increasing PKC-mediated ROS production, while antioxidant therapy reversed the expression implying an important role of ROS in the pathogenesis of podocyte injury in diabetic renal disease [238].

At initial stage, though VEGF increases filtration rate accompanied by microalbuminuria via enhanced neoangiogenesis, its subsequent reduction resulting from increased podocyte loss during progressive period of the disease eventually diminishes GFR [239]. This is supported by increased urinary excretion of VEGF and its high mRNA expression in the glomerulus during early stage of diabetic nephropathy in OLETF rats [235]. Interestingly, increased urinary VEGF levels showed a significant positive correlation with UAE and serum creatinine indicating its role in the pathogenesis of renal injury [235].

Though many studies exhibited the salutary effects of anti-VEGF agents to treat diabetic nephropathy, some other studies have shown potential complications associated with anti-VEGF treatment. Studies have found that administration of anti-VEGF neutralizing antibodies can significantly decrease hyperfiltration, albuminuria, and glomerular hypertrophy [240–242]. Furthermore, inhibition of VEGF binding with its receptor or impairment of VEGF receptor-mediated downstream signaling by sFlt-1 (soluble VEGF receptor-1) in podocytes has effectively improved diabetes-induced albuminuria, mesangial expansion, GBM thickening, podocyte foot process fusion, and TGF-*β* expression in diabetic mice [243]. In agreement with this study, Sung et al. [244] showed that inhibition of VEGF receptor phosphorylation through blocking the VEGF-tyrosine kinase activity ameliorated albuminuria, GBM thickening, and restored nephrin levels in diabetic mice. It is interesting to note that some collagen derivatives such as tumstatin (cleavage product of collagen IV) and endostatin (cleavage product of collagen XVIII) have been reported to prevent neovascularization in streptozotocin-induced diabetic mice by inhibiting some angiogenic factors including VEGF and suppressed glomerular hypertrophy, hyperfiltration, and albuminuria [245, 246]. Similarly, these peptides have decreased glomerular mesangial expansion, extracellular matrix accumulation, monocyte/macrophage deposition, TGF-*β* expression (inhibited only by endostatin), and type IV collagen expression which are potential pathological events induced in diabetic nephropathy. However, both peptides significantly increased nephrin expression in podocyte maintaining the integrity of slit-diaphragm leading to prevention of excess protein leakage [245, 246]. Recently, sulodexide, a compound made up of heparan and dermatan sulfate types of GAGs, has suppressed podocyte-specific VEGF synthesis through inhibition of high glucose-induced p38-MAPK in OLETE rats, a type 2 diabetic animal model, and it has elicited all aforementioned anti-VEGF agent-mediated renoprotective effects including decreased urinary albumin excretion and expression of profibrotic molecules [247]. Taken together, these results suggest that antiangiogenic therapy might be helpful in maintaining the glomerular barrier, resulting in the amelioration of albuminuria and other nephrotic syndromes.

In contrast to these renoprotective effects, many investigations found deleterious effects associated with anti-VEGF therapy for neoplastic diseases. These deleterious effects may include but are not limited to proteinuria, hypertension, and thrombotic microangiopathy [248, 249]. For instance, cancer patients treated with bevacizumab, a humanized monoclonal antibody against VEGF, experienced aggravated pathological events including proteinuria, hypertension, extensive foot process effacement, and thrombotic microangiopathy [250]. Administration of anti-VEGF agent, mutation, or gene deletion of podocyte-specific VEGF in murine models also exhibited similar adverse consequences. In addition, some studies have shown a beneficial role of VEGF which includes the prevention of progressive capillary rarefaction, promotion of capillary repair, improvement of renal injury, and prevention of functional and histologic abnormalities in diabetic nephropathy [250, 251]. In support of this evidence, Oltean et al.'s [251] transgenic podocyte-specific overexpression of VEGF-A165b in streptozotocin-induced diabetic mice demonstrated less glomerular hypertrophy, less mesangial expansion, and less GBM thickening. Similarly, systemic administration of VEGF-A165b in streptozotocin-induced diabetic mice improved proteinuria and GBM thickening but not mesangial expansion [251]. Based on these studies, it is obvious that VEGF expression should be optimum in renal cells, imbalance of which triggers injurious effects manifested by nephrotic syndromes and cardiovascular abnormalities.

#### 7.6.3. Connective Tissue Growth Factor (CTGF)

CTGF is an important downstream mediator of TGF-*β*1 signaling cascade and executes profibrotic as well as hypertrophic functions of TGF-*β*1 [252, 253]. Therefore, CTGF plays a pivotal role in TGF-*β*1-induced ECM production which causes mesangial expansion and increased GBM thickness leading to glomerulosclerosis and interstitial fibrosis, the progressive stage of renal injury [254, 255]. It also induces hypertrophy of mesangial cells through activation of p21^Cip1^ and p27^kip1^ and causes functional impairment as well as loss of podocytes resulting in diminished regulatory functions of the glomerulus [253, 256]. In addition, CTGF can activate proinflammatory signaling molecule NF-*κ*B which in turn upregulates various chemokines (e.g., MCP-1 and ICAM-1) and cytokines (e.g., IL-6 and IL-4) leading to increased interstitial infiltration of immune cells such as monocytes/macrophages and/or T cells to worsen renal injury [257]. In diabetic condition, CTGF is upregulated in mesangial cells and podocytes to advance its fibrotic process which is aggravated by CTGF-mediated inhibition of matrix degradation through increased production of TIMPs (tissue inhibitor of metalloproteinases) [258].

#### 7.6.4. Platelet-Derived Growth Factor (PDGF)

PDGF is a cytokine that is involved in mediating and modulating many biological processes which occurred during renal injury. PDGF mediates its diverse effects, including proliferation, differentiation, extracellular matrix accumulation, tissue permeability, pro- as well as anti-inflammatory mediators, and migration of mesenchymal cells. It evokes its actions by interacting with its receptor, PDGFR, which can be expressed on mesenchymal, mesangial, and glomerular endothelial cells. PDGF is also important for physiological angiogenesis by the recruitment of perivascular cells, for example, pericytes, and it regulates vascular tone and platelet aggregation. PDGF binding with its receptor can trigger many signaling pathways, for example, Ras-MAPK, JAK/STAT, PLC-*γ*, and PI3K pathways, to induce transcription of genes involved in proliferation, migration, and survival. In renal injury, PDGF causes pronounced mesangial cell proliferation resulting in mesangioproliferative nephritis and renal interstitial fibrosis. PDGF-mediated stimulation of MC also promotes increased expression of many inflammatory mediators, including TGF-*β*1, PAI-1, IL-6, endothelin-1, and iNOS to increase extracellular matrix production, intraglomerular pressure, and vascular resistance, thus reducing renal blood flow as well as GFR [257, 259, 260].

#### 7.6.5. Adhesion Molecules

Adhesion molecules such as ICAM-1 (intercellular adhesion molecule-1) and VCAM-1 (vascular cell adhesion molecule-1) play crucial role in infiltration of immune cells to endothelium, mesangium, and GBM. Invasion of immune cells (leukocytes) follows few steps: cell tethering, selectin-mediated rolling of cell on the endothelium, chemokine-dependent integrin activation and leukocyte adhesion, and finally transmigration of leukocytes across the endothelium. Interestingly, these processes can be advanced by the help of any adhesion molecules mentioned above to initiate immune response in local tissues [261].

ICAM-1 is a cell surface glycoprotein belonging to Ig superfamily and binds to *β*_2_ integrins, such as lymphocyte function-associated antigen-1 (LFA-1) and macrophage-1 antigen (Mac-1), which are located on most leukocytes, thereby helping leukocytes to firmly attach to the endothelium. ICAM-1 is upregulated in response to certain kinds of stimuli, such as proinflammatory cytokines (e.g., TNF-*α* and IL-1), high glucose, AGEs, oxidative stress, shear stress, and protein kinase C activation [262, 263]. In addition, ICAM-1 expression is also upregulated in both type 1 [264] and type 2 [265] models of diabetic nephropathy accompanied by disease progression. In order to ascertain damaging role of ICAM-1 in type 2 diabetic nephropathy, Chow et al. [266] evaluated the development of renal injury in both ICAM-1 intact and deficient db/db mice with similar glucose level and obesity and found that ICAM-1 deficient db/db mice showed significantly attenuated glomerular hypertrophy and renal fibrosis accompanied by reduced glomerular and interstitial infiltration of macrophages. Similarly, Okada et al. [267] showed that ICAM-1 knock-out mice have been able to prevent the progression of albuminuria, glomerular infiltration of macrophages, glomerular hypertrophy, and interstitial fibrosis at 6 months after the induction of diabetes mellitus, whereas ICAM-1^+/+^ demonstrated the opposite outcome. However, degree of albuminuria between ICAM-1-null mice and wild type mice was not different at 1 month after the injection of streptozotocin suggesting noninvolvement of ICAM-1 in increased albuminuria in the early stages of diabetic renal injury. Taken together, it is evident that OCAM-1-mediated inflammation observed in the diabetic kidney probably contributes to the progression of the disease rather than its onset.

VCAM-1, a member of Ig superfamily, is also a cell surface protein expressed on endothelial cells and some leukocytes such as macrophages and helps in their adhesion. It has been reported to be overexpressed on endothelial cells and infiltrating leukocytes in renal interstitium in diabetic animal models. In type 2 diabetes, serum level of VCAM-1 is likely to be increased and it positively correlates with albuminuria [262]. VCAM-1 expression is increased in response to several stimuli, including TNF-*α*, IFN-*γ* [268], high glucose, AGEs, oxidative stress, and Ang II [269].

### 7.7. Chemokines

Chemokines are small cytokines that are secreted by cells/leukocytes to induce recruitment of leukocytes to nearby host cells. They are induced and activated by primary proinflammatory mediators, for example, IL-1 and TNF-*α*. There are some common chemokines, such as MCP-1, MIP-1 *α*/*β*, and RANTES, which play crucial role in vascular and renal inflammation. They are briefly discussed below.

#### 7.7.1. Monocyte Chemotactic Protein-1 (MCP-1)

This is a potent chemokine belonging to CC chemokine family that is also recognized as chemokine (C-C motif) ligand 2 (CCL2). MCP-1 plays a key role in migration of monocytes, T cells, and macrophages to the diabetic kidney. In diabetic nephropathy, MCP-1 can be excessively produced by both inflammatory and renal resident cells which in turn induce progressive glomerular and tubule-interstitial injury by increasing macrophage infiltration. Its increased expression in type 2 diabetes is confirmed by its elevated urinary excretion accompanied with progressive tubulointerstitial damage [270]. It has been reported that MCP-1 is upregulated in response to high glucose concentrations, AGEs, oxidative stress, protein kinase C, and Ang II. Increased MCP-1 level in urine has been positively correlated with albumin excretion. However, diabetic MCP-1-null mice reduced macrophage infiltration and progression of diabetic renal injury [271, 272]. Based on these observations, it is evident that hyperglycemia-induced overexpression of MCP-1 eventually causes more advanced damage to the kidney.

In addition, macrophage inflammatory protein-1*α* (also known as CCL3) and CCL5/RANTES (regulated on activation, normal T cell expressed and secreted) are also upregulated in diabetic kidney. Increasing evidence shows that MIP-1*α* is overproduced and functionally activated to induce migration of T cells and macrophages to the kidney during diabetic and nondiabetic chronic kidney diseases [273, 274]. MIP-1*α* is increased in urine of patients with crescentic glomerulonephritis, whereas its cognate receptors, CCR1 and CCR5, are expressed In CD3^++^ T cells and CD 68^+^ macrophages which infiltrate the glomeruli and interstitium. CCR5 acts as receptor for several ligands including MIP-1*α*, MIP-1*β*, and RANTES and its activation correlates with the recruitment of T cells and monocytes, whereas deletion of this receptor does not decrease but increases the degree of inflammatory cells' infiltration because of increased interaction of ligands with still intact CCR1 receptor. However, deletion of blockade of CCR1 significantly attenuates renal chemokine expression, T cell infiltration, and glomerular crescent formation in CCR5 knock-out mice. This suggests the functional importance of CCR1 receptor and its all ligands in ultimate renal injury [273].

### 7.8. Vasoactive Substances

These are circulating substances that regulate vascular tone and systemic as well as local blood pressure. Among many, angiotensin II and endothelin have been reported to be increased in response to high glucose, ROS, and AGEs in diabetic kidney and impair structural and functional integrity of the glomerulus.

#### 7.8.1. Angiotensin II (Ang II)

Ang II not only increases vasoconstriction of glomerular capillary followed by intraglomerular pressure but also elicits more progressive pathological change to the glomerulus and renal tubules. Increasing evidence of experimental and clinical studies has shown injurious effects of Ang II during progressive kidney injury that ranges from vascular and mesangial cell proliferation, hypertrophy, podocyte apoptosis, and increased synthesis of matrix forming proteins to eventual glomerular and tubular sclerosis by induction of profibrotic mediators, namely, TGF-*β*, and various cytokines and by stimulation of fibroblasts, chemokines, and oxidative stress. Ding et al. [275] showed direct apoptosis of podocytes in culture medium treated with Ang II through activation of TGF-*β* and its downstream proapoptotic molecules and the apoptotic effect is mediated through AT1R. Ang II also accelerates nephrin loss from podocytes and induces progressive proteinuria and glomerulosclerosis which are attenuated by ACE inhibitors [276]. Together, these observations suggest that Ang II plays a key role in podocyte apoptosis and its depletion followed by proteinuria and glomerulosclerosis.

An example of damage inflicted by Ang II is matrix protein synthesis in mesangial cells. Kagami et al. [277] has shown that in vitro cell culture of mesangial cells with Ang II induces ECM accumulation via TGF-*β*-dependent mechanism. Moreover, Ang II and high glucose concentration induced mesangial cell proliferation and ECM deposition through induction of activator protein-1 (AP-1) [278], suggesting an obvious role of Ang II in progression of renal damage toward renal failure. Interestingly, to make matter worse, Ang II can also induce ROS generation through activation of NADPH oxidase system and ROS in turn enhances profibrotic effects of Ang II and podocyte apoptosis, thereby aggravating the injury through ROS-dependent activation of a complex network of signaling pathways (Figure 4) [279–281]. Blockade of angiotensin II type I receptor (AT1R) or angiotensin converting enzyme inhibitor has shown promising improvement in chronic hyperglycemia-induced renal injury.

On the other hand, endothelin-1 is a potent vasoconstrictor that is highly produced in diabetic kidney. In addition to its vasoconstriction effect, endothelin-1 can induce proteinuria, proinflammatory mediators, ECM accumulation, and infiltration of macrophages in kidney of streptozotocin-induced diabetic rats [282]. It can also promote hypertrophy, proliferation, and ROS formation in diabetic milieu. Endothelin-1 mediates all of its effects through endothelin type A (ET_A_) receptor, blockade of which reduces all these pathological events restoring normal function and structural integrity of kidney [282, 283]. In consistency with these observations, another study showed increased expression of proinflammatory cytokines (e.g., IL-6) and chemokines (MCP-1) in mesangial cells accompanied with increased collagen synthesis leading to ECM remodeling and renal fibrosis [284].

## 8. Advanced Renal Damage/ESRD

At the outset of diabetes, though renal injury is triggered by ROS-mediated loss of podocyte to a certain threshold level following microalbuminuria, major structural and functional changes occur in progressive stage which are induced by activation of diverse mediators and their signaling pathways. Major progressive pathological changes that have already been discussed include increased mesangial expansion, ECM deposition, hypertrophy and proliferation of mesangial cells, increased apoptosis of podocytes beyond threshold level, increased GBM thickening resulting from matrix forming protein deposition and expression of TIMPs, glomerular sclerosis that may have a nodular appearance (classic Kimmelstiel-Wilson nodules), inflammatory cell infiltration, and tubulointerstitial fibrosis (Figure 4). All these effects impair cross-talk among glomerular components which further exacerbates the functional and structural integrity of the whole glomerulus. This stage also induces severe renal tubular damage leading to even severe loss of nephron.

Moreover, denuded GBM which has already been left by increased podocytes depletion is no longer able to resist glomerular hydrostatic pressure allowing the GBM to be stretched to come in contact with the parietal cells of Bowman's capsule resulting in synechiae formation through capillary tuft adhesion to Bowman's capsule (adhesion of capillary basement membrane with Bowman's capsule). This tuft adhesion further degenerates the remaining podocytes located at the flanks of an adhesion leading to more podocyte loss that invokes excessive protein leakage that is termed “overt proteinuria” (macroalbuminuria) [285].

Progressively increased tubular protein load in tubular filtrate appears to keep the renal tubule under continuous challenge that results from its sustained exposure to diverse bioactive molecules including proteins. It is assumed that excessive proteins in the tubular infiltrate may elicit proinflammatory and profibrotic effects that directly contribute to chronic tubulointerstitial damage. This is initiated through the interaction of filtered proteins with proximal tubular cells, which excrete increased chemokines (e.g., MCP-1, RANTES, and complement component 3), profibrotic molecules (e.g., TGF-*β*), vasoactive substances (e.g., endothelin and Ang II), and cytokines (e.g., TNF-*α*), resulting in leukocytes infiltration, inflammation, myotransformation of interstitial fibroblasts, fibrosis, tubular atrophy, and apoptosis. Leukocyte such as macrophage migration to the tubulointerstitium can further promote production of TGF-*β*, endothelin, and Ang II exhibiting sustained profibrotic and proapoptotic effects. Moreover, imbalanced local production of endothelin, Ang II, and NO in tubules and peritubular capillary decreases peritubular capillary plasma flow and causes rarefaction of postglomerular capillaries, resulting in local hypoxia and tubular atrophy leading to increased nephron loss. Furthermore, loss of nephron can also be accelerated due to obstruction of urinary flow along the distal tubule by protein casts formed from protein overload leading to exacerbation of tubulointerstitial damage [286–289]. Together, these pathological events eventually lead to end-stage renal damage (ESRD).

As a result, at a point, kidney mass greatly reduces resulting in gradual decrease in glomerular blood flow and filtration rate. A limited number of nephrons also receive higher workload necessitating higher filtration pressure which can further weaken the attachment of podocytes to GBM. These are also complicated by increased mesangial expansion that reduces the filtration surface, thereby significantly reducing filtration rate and increasing intraglomerular pressure. Reduction of GFR can be used to represent severity of renal injury. For example, in the patient without kidney disease, GFR usually remains >90 mL/min/1.73 m^2^. However, GFR reduces to the range between 59 and 30 mL/min/1.73 m^2^ during moderate renal failure which further comes down to 15–29 mL/min/1.73 m^2^ in patients with severe renal failure. Moreover, GFR having <15 mL/min/1.73 m^2^ indicates end-stage renal damage requiring either dialysis or kidney transplantation [290]. The latter stage is achieved if the progressive signaling cascades are not intervened with pharmacological agents.

## 9. Conclusion

Chronic hyperglycemia is one of the most important risk factors for progressive renal damage. Patients having diabetes are more likely to develop microalbuminuria (proteinuria) that is used as a marker for abnormal renal function. High glucose plays pivotal role in causing abnormal renal function through stimulation of ROS generation. Increasing body of evidence shows that ROS is elevated in diabetic milieu both in vivo and in vitro. ROS are considered as important second messengers for different signaling pathways which maintain necessary biochemical interactions for the functions and survival of the tissues. However, accumulation of ROS resulting from their imbalanced generation and neutralization promotes diverse aberrant signaling pathways. Abnormal signaling in the kidney causes functional and structural changes of the glomerulus which is the center for renal damage. Though being generated from many sources, ROS originated from mitochondria and NADPH oxidase are thought to cause the onset of albuminuria followed by progression of renal damage through podocyte depletion.

It is assumed that all the components of glomerular filtration barrier remain under persistent strain in oxidative stress environment. But many studies have attributed initial renal damage to highly sensitive podocytes (visceral epithelial layer) that undergo apoptosis, foot process effacement, and detachment in high glucose-induced ROS environment. Accumulation of ROS in hyperglycemic ambience activates proapoptotic signaling pathways through upregulation and activation of p38-MAPK and caspase-3 which are downstream mediators of TGF-*β*. Increased TGF-*β* levels can also promote apoptosis through elevated production of SMAD7 (independent of p38-MAPK and caspase-3) and SMAD2/3 (via caspase-3 activation) (Figure 3) [135, 291]. Moreover, a recent study showed that increased TGF-*β*1 levels can stimulate expression of cytosolic cathepsin L (CatL) via nuclear translocation of dendrin from SD diaphragm of podocytes lacking CD2AP protein. Cytosolic CatL in turn causes reorganization of the actin cytoskeleton by proteolytically processing dynamin and synaptopodin. Alteration in the actin cytoskeleton renders podocytes hypersensitive to proapoptotic signals leading to their accelerated death [292]. Hyperglycemia can also facilitate podocyte detachment from GBM by downregulating *α*3*β*1 integrin that helps podocyte attach to GBM. High glucose levels can also attenuate expression of some slit diaphragm proteins such as nephrin, podocin, P-cadherin, CD2AP, and ZO-1 through diverse mechanisms leading to foot process effacement followed by proteinuria.

**Figure 5:**
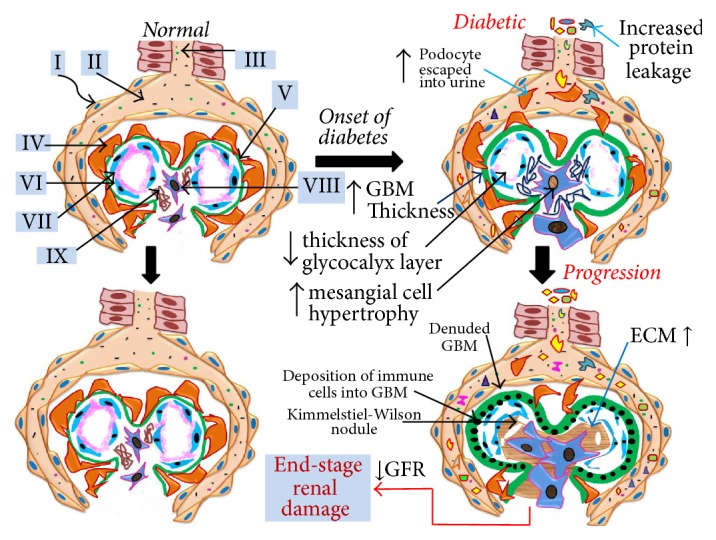
Comparison between normal and diabetic glomeruli with regard to pathological events which occurred during onset and progression of diabetes. I, parietal epithelial cells; II, Bowman's capsule; III, primary urine majorly containing water, urea, electrolytes, glucose, and so forth; IV, podocyte; V, glomerular basement membrane (GBM); VI, endothelial cells; VII, glycocalyx layer; VIII, mesangial cells; IX, extracellular matrix (ECM) proteins.

Increased albuminuria from the compromised functions of glomerular filtration barrier sets the platform for excessive activation of diverse signaling molecules. Among many, we have discussed transcription factors, inflammatory agents, growth factors, cytokines, chemokines, and vasoactive molecules in this paper in detail. Dysregulation of these abnormal signaling molecules advances the renal injury from progression of abnormal renal hemodynamics, increased glomerular basement membrane (GBM) thickness, mesangial expansion, extracellular matrix accumulation, interstitial fibrosis, and glomerulosclerosis to eventual end-stage renal damage. Lack of pharmacological intervention during progression of abnormal functional and histological change of the glomerulus may evoke irreversible end-stage renal damage which is marked by invasion of excess immune cells, classic Kimmelstiel-Wilson nodule and critically decreased glomerular filtration rate (<15 mL/min/1.73 m^2^) (Figure 5). To understand the complex signaling pathways involved in renal damage, more studies are required to uncover hidden role of glucose, ROS, and ROS-generating components in causing pathological propagation.
